# Silicon Oxynitrophosphide Nanoscale Coating Enhances Antioxidant Marker‐Induced Angiogenesis During in vivo Cranial Bone‐Defect Healing

**DOI:** 10.1002/jbm4.10425

**Published:** 2021-03-18

**Authors:** Felipe A do Monte, Neelam Ahuja, Kamal R Awad, Zui Pan, Simon Young, Harry KW Kim, Pranesh Aswath, Marco Brotto, Venu G Varanasi

**Affiliations:** ^1^ Department of Bioengineering University of Texas at Arlington Arlington TX USA; ^2^ Center for Excellence in Hip Disorders Texas Scottish Rite Hospital Dallas TX USA; ^3^ Bone‐Muscle Research Center University of Texas at Arlington Arlington TX USA; ^4^ Department of Materials Science and Engineering University of Texas at Arlington Arlington TX USA; ^5^ Department of Oral and Maxillofacial Surgery The University of Texas Health Science Center at Houston, School of Dentistry Houston TX USA; ^6^ Department of Orthopedic Surgery University of Texas Southwestern Medical Center at Dallas Dallas TX USA

**Keywords:** ANGIOGENESIS, AMORPHOUS SILICON OXYNITROPHOSPHIDE, ANTIOXIDANTS, BONE HEALING, OXIDATIVE STRESS

## Abstract

Critical‐sized bone defects are challenging to heal because of the sudden and large volume of lost bone. Fixative plates are often used to stabilize defects, yet oxidative stress and delayed angiogenesis are contributing factors to poor biocompatibility and delayed bone healing. This study tests the angiogenic and antioxidant properties of amorphous silicon oxynitrophosphide (SiONPx) nanoscale‐coating material on endothelial cells to regenerate vascular tissue in vitro and in bone defects. in vitro studies evaluate the effect of silicon oxynitride (SiONx) and two different SiONPx compositions on human endothelial cells exposed to ROS (eg, hydrogen peroxide) that simulates oxidative stress conditions. in vivo studies using adult male Sprague Dawley rats (approximately 450 g) were performed to compare a bare plate, a SiONPx‐coated implant plate, and a sham control group using a rat standard‐sized calvarial defect. Results from this study showed that plates coated with SiONPx significantly reduced cell death, and enhanced vascular tubule formation and matrix deposition by upregulating angiogenic and antioxidant expression (eg, vascular endothelial growth factor A, angiopoetin‐1, superoxide dismutase 1, nuclear factor erythroid 2‐related factor 2, and catalase 1). Moreover, endothelial cell markers (CD31) showed a significant tubular structure in the SiONPx coating group compared with an empty and uncoated plate group. This reveals that atomic doping of phosphate into the nanoscale coating of SiONx produced markedly elevated levels of antioxidant and angiogenic markers that enhance vascular tissue regeneration. This study found that SiONPx or SiONx nanoscale‐coated materials enhance antioxidant expression, angiogenic marker expression, and reduce ROS levels needed for accelerating vascular tissue regeneration. These results further suggest that SiONPx nanoscale coating could be a promising candidate for titanium plate for rapid and enhanced cranial bone‐defect healing. © 2020 The Authors. *JBMR Plus* published by Wiley Periodicals LLC. on behalf of American Society for Bone and Mineral Research.

## Introduction

Loosening and failure of fixative implants in bone‐reconstruction surgeries is unfortunately common and can be attributed to a variety of factors such as delayed healing and lack of osseointegration. Surgical revisions after these complications can be twice as expensive as the initial procedure and jeopardize functional and aesthetic outcomes.^(^ ^1^ ^)^ Craniofacial and orthopedic implant failure is defined as the inability of the implant/plate to osseointegrate with the surrounding host tissue based on a foreign body response and/or an opportunistic infection.^(^ ^2^, ^3^ ^)^ During the first few days of biomaterial implantation in the bone defect, the surrounding biological tissue can face severe and harmful oxidative stress conditions.^(^ ^2^, ^3^, ^4^ ^)^ High levels of oxidative stress can be deleterious to endothelial cells.^(^ ^5^ ^)^ Endothelial cell survival and proliferation is necessary for adequate new blood vessel formation and thus plays a vital role in bone regeneration.^(^ ^6^ ^)^ Therefore, to minimize complications, such as implant/plate–screw device loosening and failure, and to improve healing rates, the material used for bone‐defect reconstruction must have innate antioxidant stimulus, as well as the ability to enhance angiogenesis. Silicon has been used in combination with nitrogen and/or oxygen for implant/plate device composition and coating to enhance new bone formation and osseointegration.^(^ ^7^, ^8^, ^9^, ^10^ ^)^

A recent study by our group found that ionic Si is important for superoxide dismutase 1 (SOD‐1) activity.^(^ ^9^ ^)^ The enzyme SOD‐1 plays a major role in the metabolism of superoxide, which is the main ROS produced in cell metabolism. Moreover, ionic silicon can enhance angiogenesis by upregulating hypoxia inducible factor‐1 alpha (HIF‐1α) and vascular endothelial growth factor A (VEGFA) in human umbilical vein endothelial cells (HUVECs).^(^ ^11^ ^)^ Studies have found that ionic phosphorus can enhance angiogenesis by upregulating VEGFA and inducing cell migration, matrix deposition, and capillary tubule formation in HUVECs.^(^ ^12^, ^13^, ^14^ ^)^ Yet, there has not been any evidence linking biomaterials that release factors that can stimulate a key angiogenic marker, angiopoietin‐1 (ANG‐1), under toxic oxidative stress conditions. ANG1 is a key marker that, when expressed, prevents apoptosis in endothelial cells under critical survival conditions by activating the receptor tyrosine kinase Tie2.^(^ ^15^, ^16^, ^17^, ^18^ ^)^

In our earlier work, we found that plasma‐enhanced chemical vapor deposition‐ (PECVD‐) coated silicon oxynitride‐ (SiONx‐) and silicon oxynitrophosphide‐ (SiONPx‐) based fixative implants can upregulate ANG1 in HUVECs under standard in vitro growth conditions (no oxidative stress).^(^ ^19^ ^)^ PECVD is a coating method that has proven to be useful for biomaterial manufacturing because of its use of relatively low temperature processing, ability to be manufactured onto 3D surfaces for clinically relevant fixative implants, ability to enhance interfacial adhesion, form surface functional groups (hydroxyl and amide functional groups), and to allow surface patterning before coating, which enhances cell attachment and matrix deposition by mimicking the nanostructure and microstructure of an extracellular matrix.^(^ ^8^, ^9^ ^)^ In this study, we examine the use of PECVD SiONx‐based or SiONPx‐based materials to enhance angiogenic properties of endothelial cells under deleterious oxidative stress conditions.

Here, we hypothesize that a PECVD amorphous SiONx or SiONPx nanoscale coating will increase the expression of both antioxidant (SOD‐1, catalase‐1 [CAT‐1], nuclear factor erythroid 2‐related factor 2 [NRF2]) and angiogenic (CD31, ANG1) markers, and enhance vascular tubule formation while reducing ROS (4‐hydroxynonenal [4‐HNE], hydrogen peroxide [H_2_O_2_])_._ To test these hypotheses, we investigated the effect of PECVD nanoscale coatings formed by Si, O, N, and P gas reagent sources on angiogenesis under a toxic oxidative stress environment induced by 0.6mM H_2_O_2_, which was determined previously.^(^ ^20^ ^)^ in vitro experiments evaluated the effect on the viability of HUVECs, matrix deposition, capillary tubule formation, and gene expression of angiogenic and antioxidant markers on coated and uncoated surfaces. in vivo studies using adult male Sprague Dawley rats (approximately 450 g) were used for this experiment to measure local and systemic levels of oxidative stress markers and local angiogenic markers. The in vivo study analyzed 4‐HNE levels in the blood serum, and CD31 and 4‐HNE on a histological section after the fixative implants were placed in a rat standard sized calvarial defect.

## Materials and Methods

### PECVD SiONx‐ and SiONPx‐coated fixative plate preparation and analysis of surface elemental composition

In the current study, to test the SiONx and SiONPx nanoscale coating, 4‐inch <100> test‐grade P‐type silicon wafers (NOVA Electronic Materials, Flower Mound, TX, USA) were used after standard cleaning procedures. The reasons for using a silicon wafer as the base fixative implant substrate are (i) the ease of the fabrication process to form highly conformal, adherent, and uniform thin film overlays by PECVD, and (ii) the ease of dicing the silicon wafer into an appropriate plate size. The silicon wafers were immersed in piranha solution (3:1 mixture of sulfuric acid PH_2_SO_4_, 96%] and H_2_O_2_ (30%) for 10 minutes, and then rinsed in deionized (DI) water for 1 minute. To remove the native oxide layer, the silicon wafers were immersed in hydrofluoric acid for 30 to 60 seconds. Finally, the wafers were rinsed for three cleaning cycles under a continuous DI water purge, dried with N_2_ gas, and placed on a 200°C hot plate for 5 minutes.^(^ ^8^, ^9^ ^)^

A TRION ORION II PECVD/LPECVD system (Trion Technology, Clearwater, FL, USA) was used to deposit a 200‐nm uniform coating of SiONx and two different compositions of SiONPx overlays (SiONPx1 and SiONPx2), which have different Oxygen/Nitrogen (O/N) ratios as given in Table 1. All coatings were processed at a substrate temperature of 400°C, chamber pressure of 900 mTorr, an inductively coupled plasma power of 30 W, and an applied excitation frequency of 13.56 MHz. Gas source and flow rate of each gas are also provided in Table 1.

**Table 1:** The Three Steps and Flow Rates of the Different Gases Used for Processing SiON, SiONP1, and SiONP2 Implants

	SiH_4_/Ar (15/85%)	PH_3_/SiH_4_/Ar (2/15/83%)	N_2_O	N_2_	NH_4_	Ar	Time (s)
Step 1	0	0	0	0	0	250	30
Step 2 (SiON_x_)	24	0	155	225	50	0	226
Step 2	0	24		225	50	0	322
(SiONP_x_)1			5				
(SiONP_x_)2			16				
Step 3	0	0	0	0	0	250	30

Ar = Argon (carrier gas); N_2_ = nitrogen; N_2_O = nitrous oxide; NH_4_ = ammonia; PH_3_ = phosphine; SiH_4_ = silane; SiON = silicon oxynitride; SiONP = silicon oxynitrophosphide.

The refractive indices and film thicknesses were measured using ellipsometry at a wavelength of 632.8 nm (Gaertner LS300; Gaertner Scientific Corp., Skokie, IL, USA). The results of ellipsometry were confirmed using a reflectometer (NC‐UV–VIS TF; Ocean Optics, Largo, FL, USA).

High‐resolution SEM (Hitachi S‐3000N variable pressure; Hitachi High‐Technologies Corp., Tokyo, Japan) was used to image and analyze surface properties and verify film thickness of the coatings at a voltage of 20 KeV. SEM was also used to identify and measure the surface elemental composition of the PECVD‐coated plates using energy dispersive X‐ray analysis apparatus at a voltage of 12 KeV. The surface elemental composition is given in Table 2 and is explained in the Results section.

**Table 2:** Energy‐Dispersive X‐Ray Spectroscopy Analysis of Atomic Surface Composition of SiON, SiONP1, and SiONP2 Coating

EDS compositional data (at %)				
Sample	Si	O	N	P
SiONx	52.5	35.1	12.3	0
SiONPx1	61.8	7.3	30.5	0.28
SiONPx2	58.7	14.2	26.8	0.27

EDS Compositional data in units of (at %).

EDS = Energy‐dispersive X‐ray spectroscopy; SiON = silicon oxynitride; SiONP = silicon oxynitrophosphide.

### In vitro study

Several factors were used to help design the experimental setup. Prior studies evaluated the protective effect of a drug on HUVECs under oxidative stress. The cells were exposed to H_2_O_2_ at toxic levels (≥0.5mM) for 24 to 48 hours prior to treatment.^(^ ^21^, ^22^ ^)^ Further, a hypoxic condition of 1% O_2_ can induce HUVECs autophagy.^(^ ^23^ ^)^ If the cells are also exposed to excessive time of low fetal bovine serum (malnutrition), this can induce phenotype changes in HUVECs.^(^ ^24^ ^)^ HUVECs cannot survive or maintain their phenotype if they are not supplemented with endothelial cells growth media.^(^ ^25^ ^)^ Thus, a 48‐hour time limit of exposure of cells and ECM to H_2_O_2_ was implemented.

HUVECs (passage 2 to 4), endothelial cell growth medium 2 (EGM‐2; used for cell growth and subculture), and endothelial cell basal media 2 (EBM‐2; used for the proliferation and differentiation experiments) were acquired from Lonza (Morristown, NJ, USA). The experiments were divided into five groups: I = glass cover slip (GCS), II = tissue culture plate (TCP), III = SiONx, IV = SiONPx1 (O: 7.3 atomic percent (at. %)), and V = SiONPx2 (O: 14.2 at. %). Propidium iodide fluorescent stain (dead cells), calcein‐AM (for live cells), actin staining 488, and 4,6‐diamidino‐2‐phenylindole nuclei staining were used as fluorescent dyes on the fixed samples. Purified mouse antifibronectin (BD Bioscience, San Jose, CA, USA) as a primary antibody and goat anti‐mouse with Alexa 488 was used as the secondary antibody for fibronectin immunostaining. Quantitative data analysis of the images was done by ImageJ.^(^ ^26^, ^27^, ^28^ ^)^ Captured images were loaded onto ImageJ for Microsoft Windows for analysis. After loading, images were adjusted for brightness, contrast, and sharpness as needed. The images were then converted to 32‐bit encoding. The threshold was then adjusted until the entire extracellular matrix (ECM) was selected; using the zoom or magnification function. Then, the ECM area was measured as a percentage of the total view. Several images were captured from different areas of each sample, and an average was recorded.

H_2_O_2_ at a concentration of 0.6mM was used to mimic an oxidative stress environment, the stock solution was acquired from Sigma‐Aldrich (St. Louis, MO, USA) 30% w/w in H_2_O_2_ with stabilizer. The H_2_O_2_ has been used previously for in vitro studies^(^ ^29^, ^30^, ^31^ ^)^ as a major product of oxidative stress, mainly produced by swift conversion from superoxide.^(^ ^32^, ^33^, ^34^ ^)^ Based on previous publications, this specific H_2_O_2_ concentration has been shown to be deleterious to live tissue.^(^ ^19^, ^20^ ^)^ PBS (Sigma‐Aldrich), trypsin, buffer, trypsin neutralizer (Lonza), Angiopoietin 1 Human ELISA kit (Invitrogen, Waltham, MA, USA), and HNE‐competitive ELISA kit (Cell Biolabs, Inc., San Diego, CA, USA) were used for the in vitro experiments.

#### Cell viability under toxic hydrogen peroxide

PECVD SiONx‐ and SiONPx‐coated plates (square shape) with a dimension of 1.2 × 1.2 cm and GCS measured 1.5‐cm diameter round were used for the experiment (four samples per group). HUVECs were seeded on the plate surfaces (5000 cell/cm^2^), placed in a 12‐well plate with 100 μL of EGM‐2 according to the protocol described by Lonza, and allowed to attach on the surface for 1 hour.^(^ ^35^, ^36^ ^)^ After initial attachment 900 μL of EBM‐2 + 10% FBS + H_2_O_2_ 0.6mM was added and cultured for 24 hours. An MTS, tetrazolium cell proliferation assay kit, was performed on four samples per group for cell viability; three samples per group were stained with propidium iodide for fluorescent imaging.

#### Matrix deposition under toxic hydrogen peroxide

Cells were seeded as mentioned above along with the same media for mimicking an oxidative stress environment (four samples per group). After 48 hours under oxidative stress, the media was changed to EBM‐2 + 10% FBS without H_2_O_2_. Five days after initial cell seeding, the cells were fixed with 4% paraformaldehyde, and immunostaining was done for fibronectin using the antibodies mentioned above. Three pictures from different areas were captured per sample using ×10 magnification on a Zeiss Axion cell culture fluorescent microscope (Carl Zeiss Microscopy, Peabody, MA, USA), and ImageJ was used for quantifying the area occupied by fibronectin.

#### Capillary tubule formation under toxic hydrogen peroxide

All PECVD‐coated plates were cut to 0.5 × 0.5 cm in dimension (four samples per group). All SiONx‐ and SiONPx‐coated plates, GCS, and pipette tips were placed in a 48‐well plate at 4°C, 12 hours before the start of the experiment. Matrigel (Corning, Inc., Corning, NY, USA) without growth supplements was slowly thawed at 4°C for 12 hours. First, 100 μL of Matrigel was placed on the implant plates, GCS, and TCP; then the well plate was placed inside the cell culture incubator at 37°C, 95% humidity, and 5% CO_2_ for 30 minutes. After 30 minutes, 100 μL of EBM‐2 + H_2_O_2_ was used to seed 60,000 cells/cm^2^ on a bed of Matrigel for each sample as mentioned in a previously described protocol.^(^ ^36^, ^37^ ^)^ Finally, 6 hours after the initial cell seeding, 50 μL of calcein AM 2μM diluted in EBM‐2 was added to each well, and after 30 minutes three pictures from different areas in magnification ×10 were captured per well. The total tube length was calculated using the Angiogenesis Analyzer software (ImageJ plug‐in).

#### Gene expression of angiogenic and oxidative stress markers on HUVECs under toxic hydrogen peroxide levels

The groups used in this experiment were: TCP, SiONx, SiONPx1 (O: 7.3 at %), and SiONPx2 (O: 14.2 at %). The cells were seeded on TCP and all three scaffolds (200,000 cells per well) in a 12‐well plate, *n* = 4 per group, and EBM‐2 + 10% FBS + H_2_O_2_ 0.6mM was used as a conditioned medium. After 24 hours, the cells were lysed using TRIzol and RNA was collected, precipitated in 70% ethanol, and the RNA solution was purified using a miRNAeasy MINI kit^(^ ^38^ ^)^ from QIAGEN (Valencia, CA, USA). RNA sample concentration was normalized to 100 μg/mL and cDNA conversion was made using a Goscript Reverse Transcriptase kit from Promega Corporation (San Luis Obispo, CA, USA). A 20‐μL reaction was prepared for RT‐PCR using TaqMan Gene Expression Assay and the PCR machine (Life Technologies, Grand Island, NY, USA) was set up with the standard protocol adjusted for 50 cycles. Results were expressed relative to the housekeeping gene 18S and compared with the control (TCP), and ΔΔCT method was used for calculations for fold change. All data are represented in fold change, and statistical analyses were done using the ΔΔCT values. The following angiogenic and oxidative stress markers were studied; Angiogenic markers: VEGFA, nesprin‐2, and ANG1; oxidative stress markers: SOD‐1, CAT‐1, nitric oxide synthase 3 (NOS‐3), glutathione peroxidase 1 (GPX‐1), and nuclear factor erythroid 2‐related factor 2 (NRF2; Table 3).

**Table 3:** Gene Expression Assay TaqMan Identification

Gene	Assay identification
VEGFA	Hs00900055_m1
Ang‐1(ANGPT1)	Hs00919202_m1
Nesprin‐2 (SYNE2)	Hs00794881_m1
SOD‐1	Hs00533490_m1
Cat‐1 (CAT)	Hs00156308_m1
e‐NOS (NOS3)	Hs01574665_m1
Nrf2	Hs00975961_g1

Ang‐1 = Angiopoietin 1; Cat‐1 = catalase 1; e‐NOS = endothelial nitric oxide synthase; Nrf2 = nuclear factor erythroid 2‐related factor 2; SOD‐1 = superoxide dismutase 1; VEGFA = vascular endothelial growth factor A.

#### Angiopoietin‐1 and 4‐HNE levels in conditioned medium using ELISA

Using the same groups, sample size, and conditioned medium used for PCR (see the above section), the culture media was collected before lysing the cells for RNA extraction and placed in a 1.5‐mL centrifuge tube with protease inhibitor (1:1000 dilution) and stored at −20°C. These supernatant media samples were then used for quantification of the protein concentration by bicinchoninic acid (BCA) assay and ANG1 ELISA. The data and comparison among groups were expressed in a bar graph showing the values relative to control (TCP). The 4‐HNE was measured by competitive ELISA and expressed in a bar graph in μg/mL.

### In vivo study

The establishment of a standardized defect is critical according to the study and also age/weight of the animal. For our study, we choose the critical/standard‐sized calvarial defect model (6 × 4 mm) to study the effect of SiONPx coatings on angiogenesis, which is a widely used model in the study of cranial bone regeneration.^(^ ^39^ ^)^ These procedures are sensitive and technique‐dependent, and require adequate training as well for reproducibility. The corrective use of a blank defect (negative control) and sham (no bone defect) is also important because they serve as controls for an empty defect with no intervention and for the surgical method without creating the defect.^(^ ^40^ ^)^ In our study, we also observed the limitation of the plate fixation to the adjacent bony tissues, which were improved by the use of tissue bond (Vetbond; 3M, Saint Paul, MN, USA) and closely reflapping the skin and suturing.^(^ ^41^, ^42^ ^)^ However, we did not reapproximate the periosteum to impose the more clinically relevant situation in which cranial injuries are accompanied by periosteum loss.^(^ ^43^ ^)^

All surgical modalities, care, and treatment plans followed the ethical use of animals' protocol approved by the Institutional Animal Care and Use Committee (IACUC) at the University of Texas at Arlington (IACUC Protocol No. A18.014). Materials used in the procedure were as follows: disposable surgical blades #15, gauze, dental burs #1 and #2, PBS, templates made of silicon wafer (6 × 4 mm), isoflurane, nalbuphine, suture (Vicryl 4–0), and other basic surgical tools.

Adult male Sprague Dawley rats (approximately 450 g) were used for this experiment to measure local and systemic levels of oxidative stress markers and local angiogenic markers. The animals were randomly assigned for specific treatment. G‐power software was used for sample size calculation with 80% power, α = 0.05, and moderate effect size of 0.30. The animal study had three groups (*n* = 3 per group): I = sham, II = uncoated plate (silicon wafer with 2 nm natively grown SiO_2_ surface), and III = 200 nm SiONPx2 (O: 14.2 at %) coated plate. For group I (sham), all steps of the surgical procedure were done except creating the bone defect and implantation, thereby accounting for aspects of the surgical procedure. For groups II and III (uncoated plate and coated plate, respectively), these materials were placed into rats that had two standard‐size calvarial defects measuring 6 × 4 mm on each side of a median sagittal suture following the same technique used in a previous publication,^(^ ^9^ ^)^ despite the traditional description as 5 to 8 mm.^(^ ^39^, ^44^, ^45^, ^46^, ^47^ ^)^ A 1‐mm gap between the plate and bone side walls was made to determine the tissue regeneration on sample surfaces without conduction from the side walls of the defect.

Immediately before surgery, painkiller/sedative SR buprenorphine 0.5/1 mg/kg was injected subcutaneously. With the animal under anesthesia (2% to 3% isoflurane), an approximately 2.5‐cm‐long incision was made on an imaginary line traced over the sagittal suture, from between the eyes to the posterior, with a sterile blade. Then, the skin and periosteum were carefully elevated and spread to expose the two parietal bones. The two bone defects were created using a dental bur #1 and/or #2 of approximately the same dimensions of 6 × 4 mm ±1 mm on either side of the midline, while the surgical site and surrounding tissues were continuously irrigated with cold saline solution to prevent heating the surrounding bone and to keep the soft tissue moist. The depth of the defect was carefully made, and the loosened bone was removed so as not to damage the dura. After cleaning the defect area, the plate was placed in the defect. The right side was empty (internal control) and the left side was implanted with SiONPx2 (O: 14.2 at %) samples or silicon wafer samples (negative control). The skin was then sutured with Vicryl 4.0.

After the surgeries, rats were examined for signs of stress and any signs of pain or distress. Analgesia was administered to the animals for management of pain and distress after surgery if needed. Then 0.1 mL of blood (from the rat tail vein) using a sterile syringe was extracted every week for further testing. The experimental end point was 2 weeks after the surgery, after which the animals underwent euthanasia (>90% CO_2_ and monitored until the animal was deceased).

#### Detection of oxidative stress marker in blood serum

Blood was collected from rat tail vein (*n* = 3 per group), placed in a centrifuge tube, and centrifuged at 5000 rpm for 10 minutes. The supernatant serum was stored in −80°C until further use. The serum samples were used to measure 4‐HNE by a competitive ELISA kit (Cell Biolabs). The animals were anesthetized using 2% to 3% isoflurane and blood was collected just before the surgery, at 1 week, and 2 weeks (just before euthanizing the animals).

#### Detection of angiogenic and oxidative stress markers (serum analysis and histological analysis)

Serum samples were used to measure ANG1 (ANG1‐7 ELISA kit [Rat] Catalog #OKDD01231), NRF2 (NFE2L2 ELISA kit [Rat] Catalog #OKEH03531), and HIF‐1α (HIF1A ELISA kit [Rat] Catalog #OKEH00594) by a competitive ELISA kit acquired from Aviva Systems Biology Corp. (San Diego, CA, USA) using the manufacturer's guidelines.

After euthanasia (2 weeks after surgery), the calvarial samples were harvested using a diamond saw preserving at least 5 mm of bone and tissue from the original defect and implanted samples. All sham samples had the sagittal suture and parietal bones preserved. The samples were fixed in 4% paraformaldehyde at 4°C for 5 days and decalcified by ethylenediaminetetraacetic acid for 1 week. Half of each sample was used for frozen section and half for plastic embedding.

##### Sanderson's staining

The samples embedded in plastic were polished and cleaned using a polishing cloth, and washed with tap water and ultrasonic cleaner. Later, the samples were stained with Sanderson's stain and counterstained with acid fuchsin. Digital images were acquired using the ×10 objective of a BIOQUANT Osteoimager (BIOQUANT Image Analysis Corp., Nashville, TN, USA). These images were used to show the anatomic relationship between the bone defects and the implant plate.

#### Immunostaining (CD31 and 4‐HNE)

The samples were perfused with sucrose and embedded in an optimal cutting temperature compound for frozen sectioning, and further immunostaining was done for CD31 (endothelial cell marker) and 4‐HNE (oxidative stress marker). The frozen samples were cut at 10‐μm thickness in a coronal plane to maintain the bone defects with and without plate in the same slide. The slides were washed with PBS and blocked with 10% goat serum. After blocking, the slides were washed again with PBS, and the primary antibodies (rabbit polyclonal 4‐HNE (ab46545) 1:8000 and rabbit polyclonal CD‐31 (M20) 1:500) were placed on the slides and incubated overnight at 4°C. The slides were then introduced to the secondary antibodies Alexa Dye 594 Goat Anti‐Rabbit in 1:200 dilution, washed twice with PBS, and mounted with a cover slip for microscopic evaluation. One slide per group was used as a negative control and was not exposed to the primary antibody. The pictures were captured from the central area just above the implant plate or central fibrotic tissue (empty) of the defect to minimize the presence of neovascularization from surrounding bone. We used a ×20 objective on Zeiss Axion cell culture fluorescent microscope for acquisition of the images and captured three images per slide. The slides were obtained from three different areas (anterior, medium, and posterior). ImageJ software was used to calculate the amount of fluorescence in relation to the total area.

#### Immunostaining (ANG1, HIF‐1α, and NRF2)

The cut and polished plastic embedded samples were washed and then kept in xylene for two changes to remove the upper plastic layer for 1 minute each. The samples were then washed in descending grade alcohol (100%, 90%, 75%, 50%, 25%, and 0%) to rehydrate the samples. The samples were then kept for antigen retrieval using testicular hyaluronidase method.^(^ ^48^ ^)^ Prewarmed testicular hyaluronidase solution was prepared using 47 mL of 0.1M potassium phosphate and 3 mL of 0.1M sodium phosphate with 0.025 g of testicular hyaluronidase (H3884; Sigma‐Aldrich) and the samples were kept in the solution for 30 minutes at 37°C. The samples were then washed three times with PBS with 1% TWEEN solution for 5 minutes each and stained with primary antibodies Angiopoietin 1 Polyclonal Antibody (23302‐1‐AP; Proteintech North America, Rosemont, IL, USA) in 1:500 dilution, HIF1α Polyclonal Antibody (PA1‐16601; Thermo Fisher Scientific, Waltham, MA, USA), and NRF2/NFE2L2 Polyclonal Antibody (16396‐1‐AP; Proteintech North America) in 1:500 dilution overnight at 4°C, washed three times again, and stained with secondary antibodies Alexa Dye 594 Goat Anti‐Rabbit (red fluorescence) in 1:500 dilution and Alexa Dye 488 Goat Anti‐Rabbit (green fluorescence; A‐11008; Thermo Fisher Scientific) in 1:500 dilution at room temperature for 1 hour. The samples were washed, mounted, and imaged. Image J software was used to calculate the percentage of fluorescence in relation to the total area.

### Statistical analyses

The results were presented in box plots showing the mean, SD, and significance levels. Normality tests were done before statistical analysis to confirm normal distribution. Student's *t* test was used for comparing the difference in means between the two groups at a significance level *p* < 0.05. One‐way ANOVA (Tukey's pairwise) was used to compare means for more than two groups at a significance level *p* < 0.05. OriginPro 2017 (OriginLab Corporation, Northampton, MA, USA), Past3 (free software for scientific data analysis), and Microsoft Excel 2016 (Microsoft Corporation, WA, USA) software were used for graphics and calculations.

## Results

### Cells viability under toxic hydrogen peroxide

**Fig 1:**
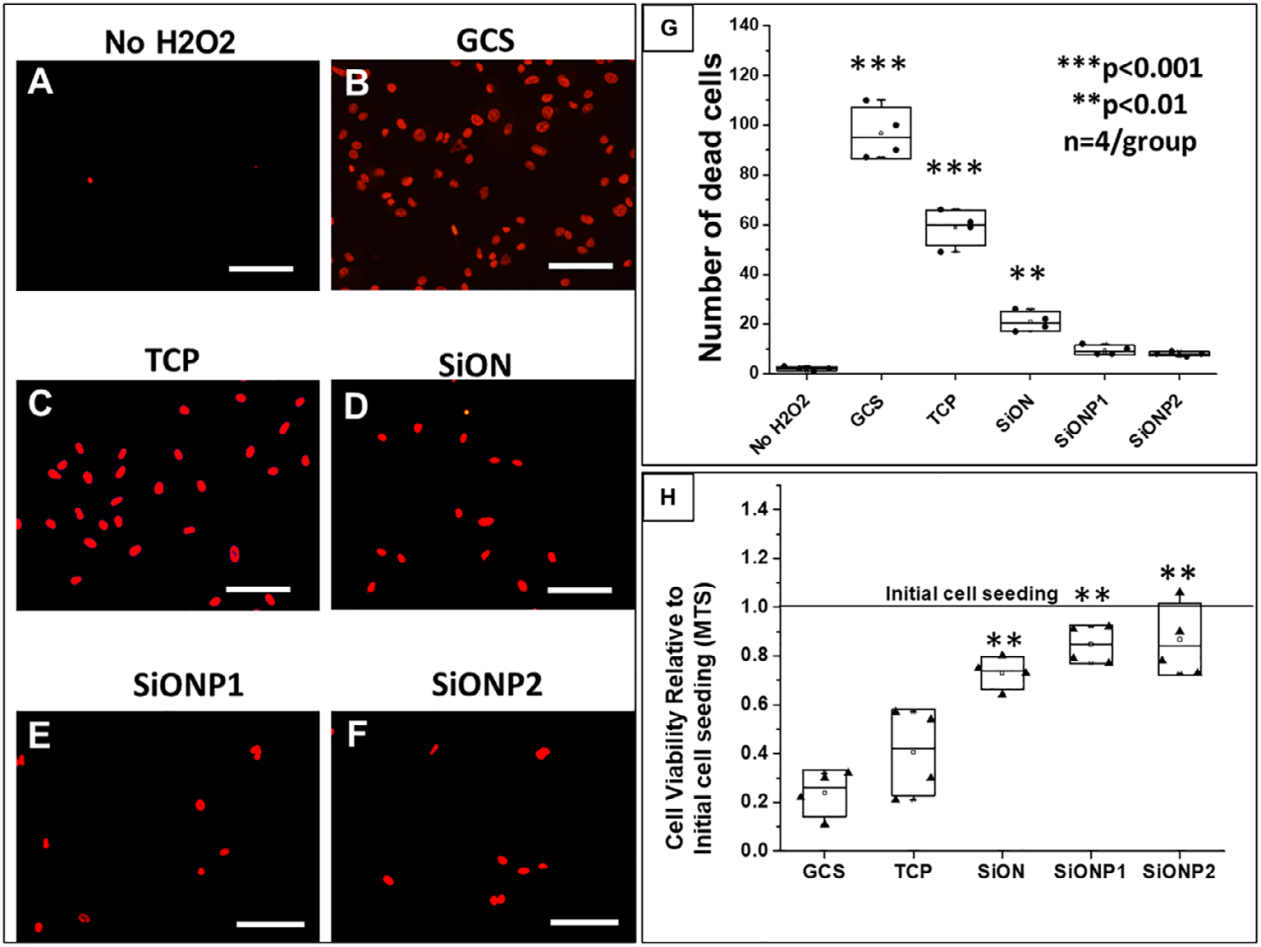
Effect of silicon oxynitride‐ (SiONx‐) and silicon oxynitrophosphide‐ (SiONPx‐) plasma‐enhanced chemical vapor deposition nanoscale implant coating on human umbilical vein endothelial cells under toxic levels of hydrogen peroxide (H_2_O_2_; 24 hours). Scale bar = 100 μm. (*A*–*F*) Propidium iodide (PI) staining shows dead cells (red stain). (*G*) Analysis after ANOVA (Tukey's pairwise) shows data from PI counting according to group. (*H*) Comparison of cell viability relative to the initial cell seeding among groups after MTS assay, the data were analyzed by ANOVA (Tukey's pairwise). GCS = glass cover slip; SiONP = silicon oxynitrophosphide; TCP = tissue culture plate. *n* = 4. ***p < 0.001 and **p < 0.01, all compared with No H_2_O_2_ group.

Figure 1 shows the cells viability study using propidium iodide fluorescent stain; the data are presented by boxplots with the statistical significance for each group. We compared the study groups as follows: No H_2_O_2_, GCS, TCP, SiON, SiONPx1, and SiONPx2. The cells in the last five groups were exposed to toxic concentrations of H_2_O_2_. The SiONPx1 and SiONPx2 groups presented a reduced number of dead cells (red stain) and were similar to the No H_2_O_2_ group (*p* > 0.05) as shown in Fig. 1(*A*–*F*). GCS and TCP showed a significant increase in the number of dead cells compared with the other groups (*p* < 0.01; Fig. 1*G*). Also, an MTS assay was used to study the cell viability on the surface of the different study groups (GCS, TCP, SiONx, SiONPx1, and SiONPx2). The data are presented in a boxplot at Fig. 1*H*. All groups were exposed to the same toxic concentration of H_2_O_2_ and relative to initial cell seeding. GCS and TCP showed a significant reduction in the number of viable cells, approximately 20% and 40%, respectively, compared with the initial cell seeding (*p* < 0.001). TCP presented an almost 50% lower number of cells compared with SiONx, SiONPx1, and SiONPx2 (*p* < 0.01). There was no significant difference among SiONx‐ and SiONPx‐coating groups (SiONx, SiONPx1, and SiONPx2; *p* > 0.05; Fig. 1*H*).

### Matrix deposition after exposure to toxic hydrogen peroxide environment

**Fig 2:**
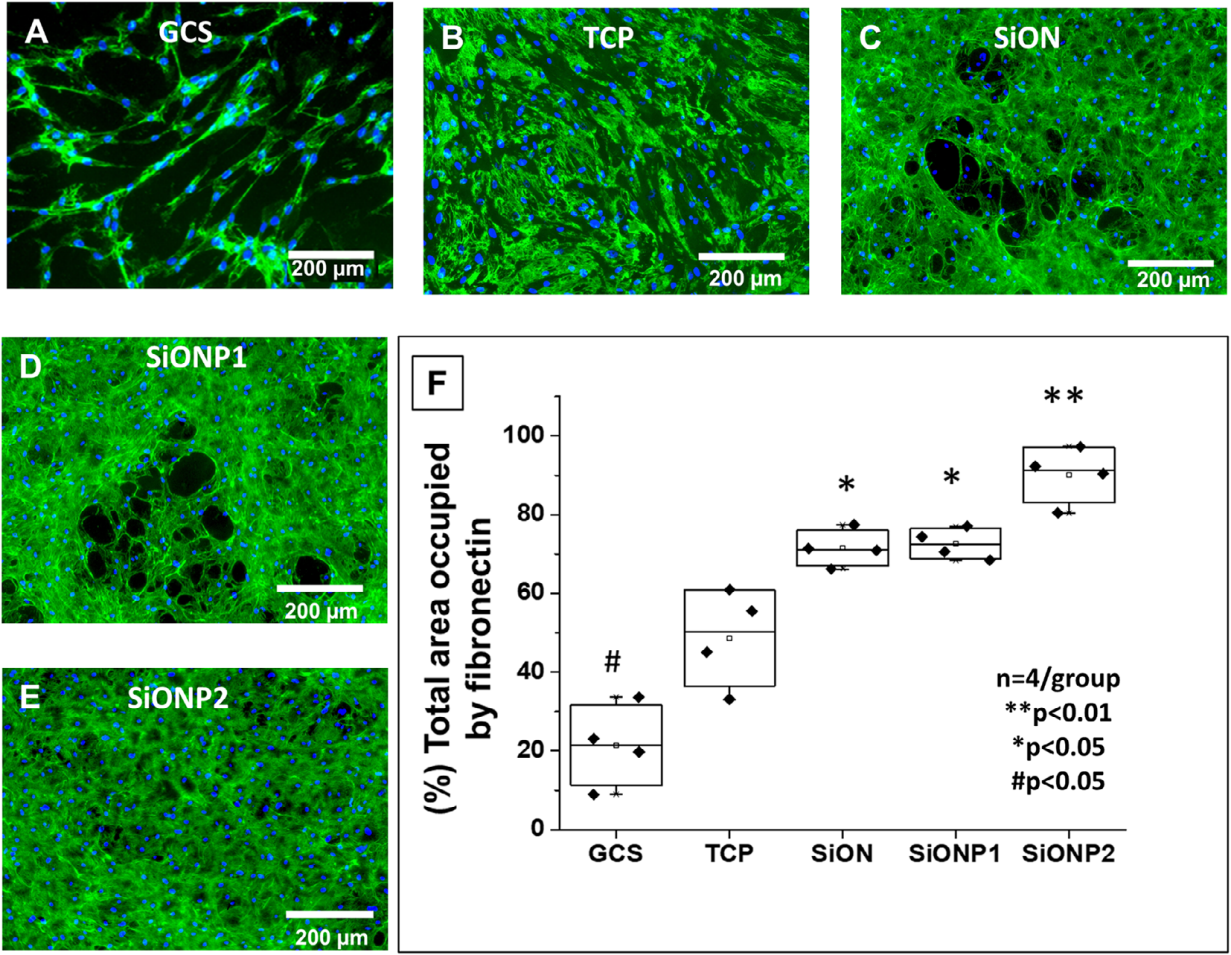
Fibronectin deposition by human umbilical vein endothelial cells (HUVECs) in vitro. (*A*–*E*) Fluorescent images after immunostaining for fibronectin deposition 5 days after HUVECs were exposed to toxic oxidative stress (0.6mM hydrogen peroxide). Scale bar = 200 μm. (*F*) Graph of data analysis after ANOVA (Tukey's pairwise) shows of percentage of area occupied by fibronectin after data collection using ImageJ. GCS = glass cover slip; SiON = silicon oxynitride; SiONP = silicon oxynitrophosphide; TCP = tissue culture plate. *n* = 4. **p < 0.01, *p < 0.05, and #*p* < 0.05, compared with TCP group.

Figure 2 presents the fibronectin deposition by HUVEC cells in vitro. The amount of fibronectin deposition was evaluated by fluorescent immunostaining, and the percentage of total area occupied by fibronectin is given in a boxplot (Fig. 2*F*). The GCS surface presented three times lower fibronectin deposition (21.35 ± 10.19%) than SiONx (71.46 ± 4.58%) and SiONPx1 (72.59 ± 3.84%) groups (*p* < 0.05), and four times lower than SiONPx2 (90.11 ± 7.06%, *p* < 0.01). The TCP surface also showed a significant reduction in fibronectin deposition (48.64 ± 12.29) compared with SiONx, SiONPx1 (*p* < 0.05), and SiONPx2 (*p* < 0.01) as presented at Fig. 2(*B*–*E*). Fluorescent images showed significantly lower fibronectin deposition on the GCS surface than all other groups, but had a tubular arrangement of the fibrous network. In contrast, TCP presented more fibronectin deposition than the GCS, with minimal tubular network formation of the fibers. SiONx, SiONPx1, and SiONPx2 surfaces had significantly more fibronectin deposition compared with GCS and TCP, along with marked enhancement in the tubular network structure on all SiONx‐ and SiONPx‐coated plates. SiONPx2 (O: 14.2 at %) produced the densest fibronectin structure along with tubular arrangement of fibers compared with all groups (Fig. 2*E*).

### Capillary tubule formation under toxic hydrogen peroxide

**Fig 3:**
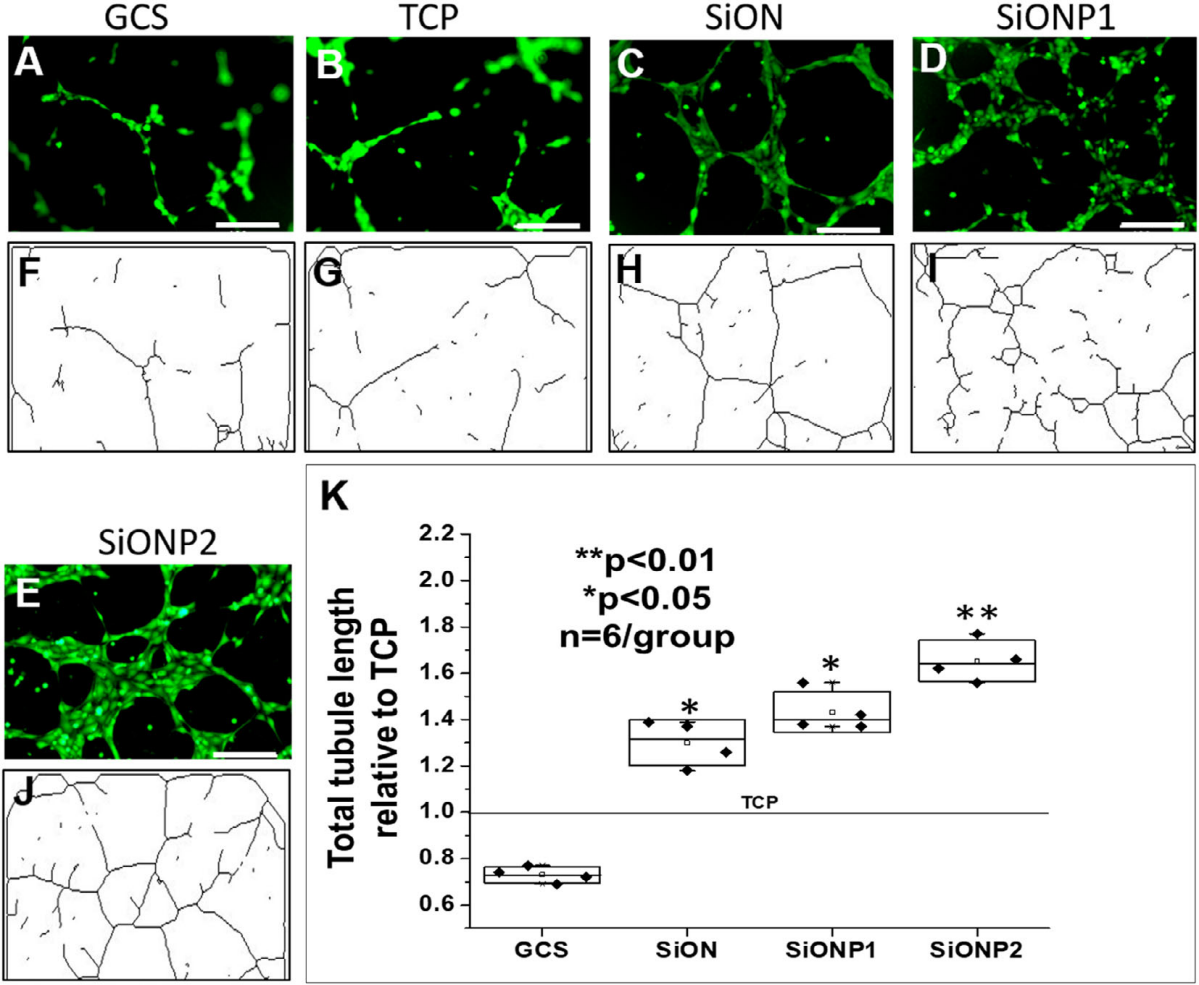
(*A*–*E*) Fluorescent images after calcein‐AM staining of human umbilical vein endothelial cells (HUVECs) capillary tubule formation under toxic oxidative stress (0.6mM hydrogen peroxide). Scale bar = 200 μm. (*F*–*J*) Tree capillary network traced lines after analysis by angiogenesis analyzer (ImageJ plugin) software. (*K*) Graph of data analysis after ANOVA (Tukey's pairwise) shows comparison of total tubule length. GCS = glass cover slip; SiON = silicon oxynitride; SiONP = silicon oxynitrophosphide; TCP = tissue culture plate. *n* = 4. **p < 0.01 and *p < 0.05, compared with TCP group.

Fluorescent images and tubular network lines traced on ImageJ software showed improved tubular structure formation and length in HUVECs placed on PECVD SiONx‐ and SiONPx‐coated materials compared with GCS and TCP (Fig. 3). All SiONx and SiONPx groups presented a significantly enhanced total tubule length (SiONx‐coated plates showed 30% [*p* < 0.05], SiONPx1 43% [*p* < 0.05], and SiONPx2 65% improvement [*p* < 0.01]) as compared with TCP. Conversely, GCS showed a significant reduction in total tubule length compared with all other groups (*p* < 0.01; Fig. 3*B,K*).

### Angiopoietin‐1 and 4‐HNE protein adduct levels in conditioned medium after oxidative stress (ELISA)

**Fig 4:**
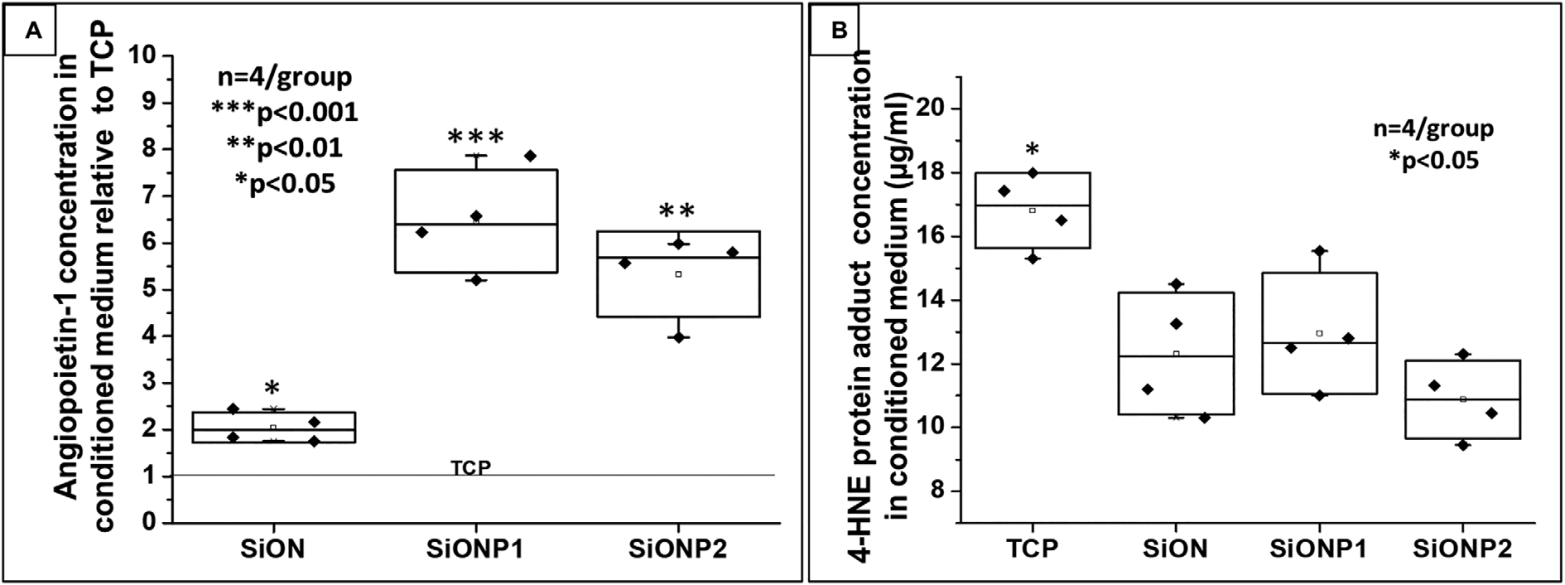
(*A*) ANG1 concentration in conditioned medium relative to TCP (control). ANOVA (Tukey's pairwise) was used for analysis and bicinchoninic acid assay was used to quantify the amount of ANG1 per μg of total protein on each sample. ***p < 0.001, **p < 0.01, and *p < 0.05, compared with TCP group. (*B*) 4‐HNE protein adduct concentration in conditioned medium. Graph of data analysis after ANOVA (Tukey's pairwise). The conditioned medium was obtained from human umbilical vein endothelial cell culture after 24 hours in toxic oxidative stress induced by 0.6mM hydrogen peroxide. *p < 0.05, compared to each of the other groups. 4‐HNE = 4‐Hydroxynonenal; ANG1 = angiopoietin 1; SiON = silicon oxynitride; SiONP = silicon oxynitrophosphide; TCP = tissue culture plate. *n* = 4.

ELISA showed that SiONx and SiONPx groups induced a marked increase in ANG1 production in HUVECs under toxic oxidative stress as compared with control (TCP) after 24 hours. The SiONx group showed twofold, SiONPx1 more than sixfold, and SiONPx2 almost fivefold increase in the levels of ANG1 (Fig. 4*A*). There was no significant difference in 4‐HNE protein adduct levels among the coated implant plate groups. However, all the SiONx‐ and SiONPx‐coated groups showed significantly reduced levels of 4‐HNE protein adduct as compared with TCP (control) (*p* < 0.05; Fig. 4*B*).

### Gene expression of angiogenic and oxidative stress markers on HUVECs under toxic hydrogen peroxide levels

**Fig 5:**
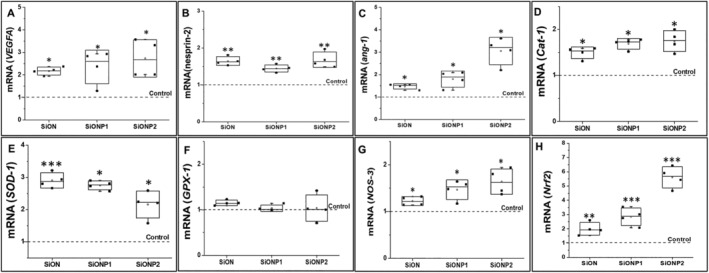
Human umbilical vein endothelial cell gene expression of angiogenic markers relative to 18S compared with control 24 hours under toxic oxidative stress (0.6mM hydrogen peroxide). (*A*) mRNA VEGFA. (*B*) mRNA nesprin‐2. (*C*) mRNA angiopoietin‐1. (*D*) mRNA Cat‐1. (*E*) mRNA SOD‐1. (*F*) mRNA GPX‐1. (*J*) mRNA NOS‐3. (*H*) mRNA Nrf2. ang‐1 = Angiopoietin 1; Cat‐1 = catalase 1; GPX‐1 = glutathione peroxidase 1; NOS‐3 = nitric oxide synthase 3; Nrf2 = nuclear factor erythroid 2‐related factor 2; SiON = silicon oxynitride; SiONP = silicon oxynitrophosphide; SOD‐1 = superoxide dismutase 1; VEGFA = vascular endothelial growth factor A. *N* = 4. ***p < 0.01 and *p < 0.05, compared with control.

All angiogenic markers (VEGFA, nesprin‐2 [NES2], ANG1) were significantly overexpressed for SiONx‐ and SiONPx‐coated plates compared with the control as shown in Fig. 5. VEGFA (Fig. 5*A*) was expressed twice as much in HUVECs seeded on PECVD‐coated plates (*p* < 0.05). NES2 (Fig. 5*B*) was overexpressed in SiONx (1.64 ± 0.11 times, *p* < 0.01), SiONPx1 (1.44 ± 0.1 times, *p* < 0.01), and SiONPx2 (1.67 ± 0.2 times, *p* < 0.01) as compared with the control group. ANG1 (Fig. 5*C*) was overexpressed in SiONx (1.47 ± 0.12 times, *p* < 0.05), SiONPx1 (1.79 ± 0.35 times, *p* < 0.05), and SiONPx2 (O: 14.2 at %; 2.71 ± 0.98 times, *p* < 0.05) more than the control group.

Among all the studied oxidative stress markers (CAT‐1, SOD‐1, GPX‐1, NRF2, NOS‐3), GPX‐1 (Fig. 5*F*) presented no significant difference compared with the control group. CAT‐1 (Fig. 5*D*) was significantly overexpressed in SiONx (1.49 ± 0.12 times, *p* < 0.05), SiONPx1 (1.69 ± 0.11 times, *p* < 0.05), and SiONPx2 (1.75 ± 0.23 times, *p* < 0.05) than the control group. SOD‐1 (Fig. 5*E*) expression was significantly more in SiONx (2.91 ± 0.24 times, *p* < 0.01), SiONPx1 (2.76 ± 0.14 times, *p* < 0.05), and SiONPx2 (2.16 ± 0.42 times, *p* < 0.05) as compared with the control group. Also, NOS‐3 (Fig. 5*G*) showed significant overexpression in SiONx (1.22 ± 0.09‐fold, *p* < 0.05), SiONPx1 (1.46 ± 0.21 times, *p* < 0.05), and SiONPx2 (1.64 ± 0.27 times, *p* < 0.05), along with the other biomarkers. NRF2 (Fig. 5*H*) was overexpressed in SiONx (1.99 ± 0.44 times, *p* < 0.01), SiONPx1 (2.85 ± 0.61, times, *p* < 0.001), and SiONPx2 (5.61 ± 0.75, times, *p* < 0.001) as compared with the control.

### In vivo evaluation of PECVD‐coated fixative plates

**Fig 6:**
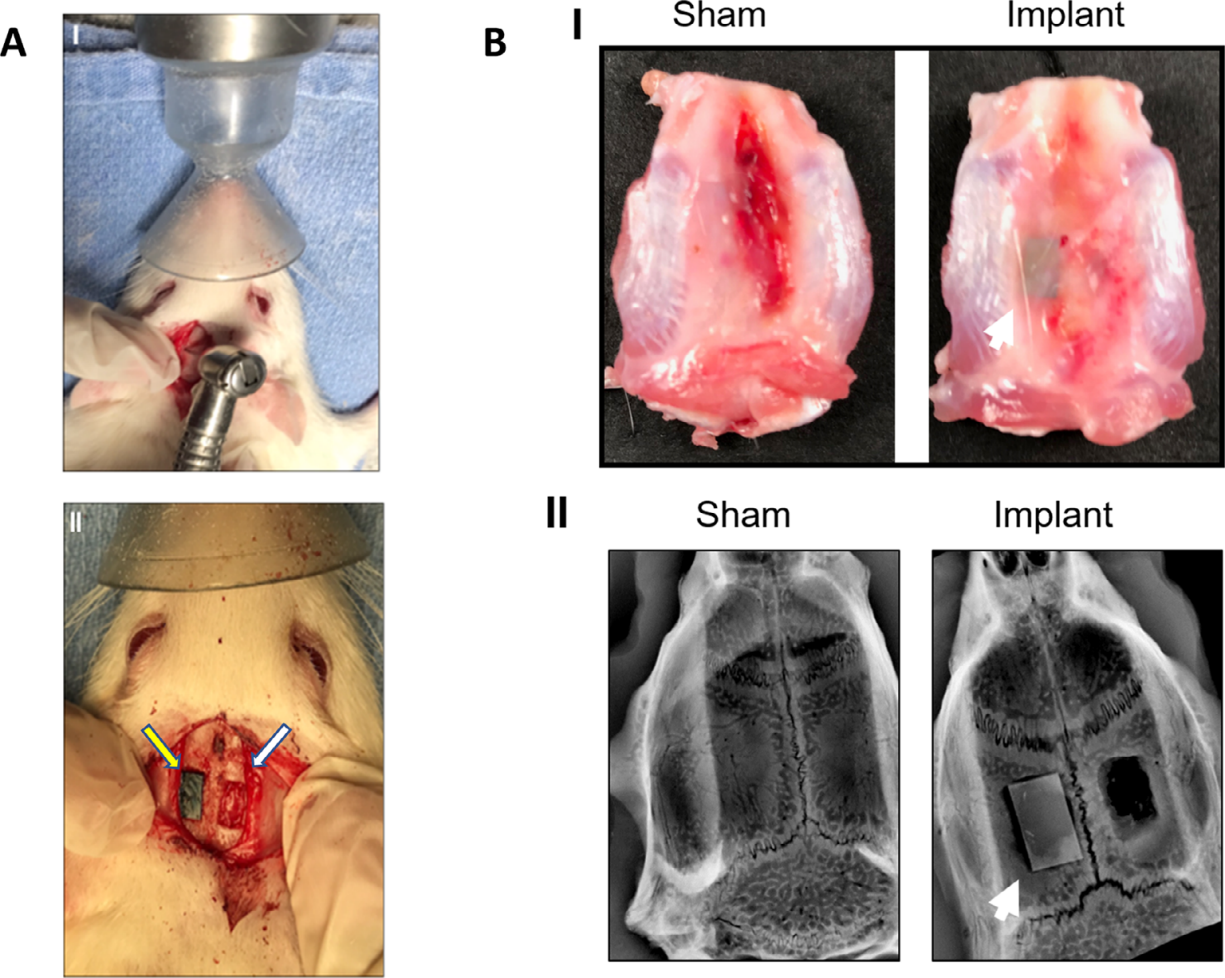
(*A*) Surgical procedure for material implantation. (I.) Gross image of calvarial defect surgery with dental bur. (II) Gross image of the parietal bone bilateral calvarial defect (6 × 4 mm), implant on the left (yellow arrow) and empty on the right (white arrow). (*B*) Samples harvested from rat calvarium 15 days after surgery. (I) Gross image shows the macroscopic superior aspect of the calvaria. (II.) An X‐ray image with the sham (left) and calvarial defects and implant (right). The white arrow points to the implant. *n* = 3.

Surgical procedures with coated and uncoated fixative implants are shown in Fig. 6*A*. During sample harvesting there was no macroscopic evidence of inflammation, and all samples were well‐positioned inside the defect as verified by X‐ray images in Fig. 6*B*. The animals were divided into three groups (*n* = 3 per group): sham, uncoated implant “Si bare wafer,” and SiONPx2. Because of the results of the in vitro experiments, SiONPx2 plate was used in the in vivo studies. It is also important to mention that no adverse events were observed with the current model and the used biomaterials.

#### Serum analysis (ELISA)

**Fig 7:**
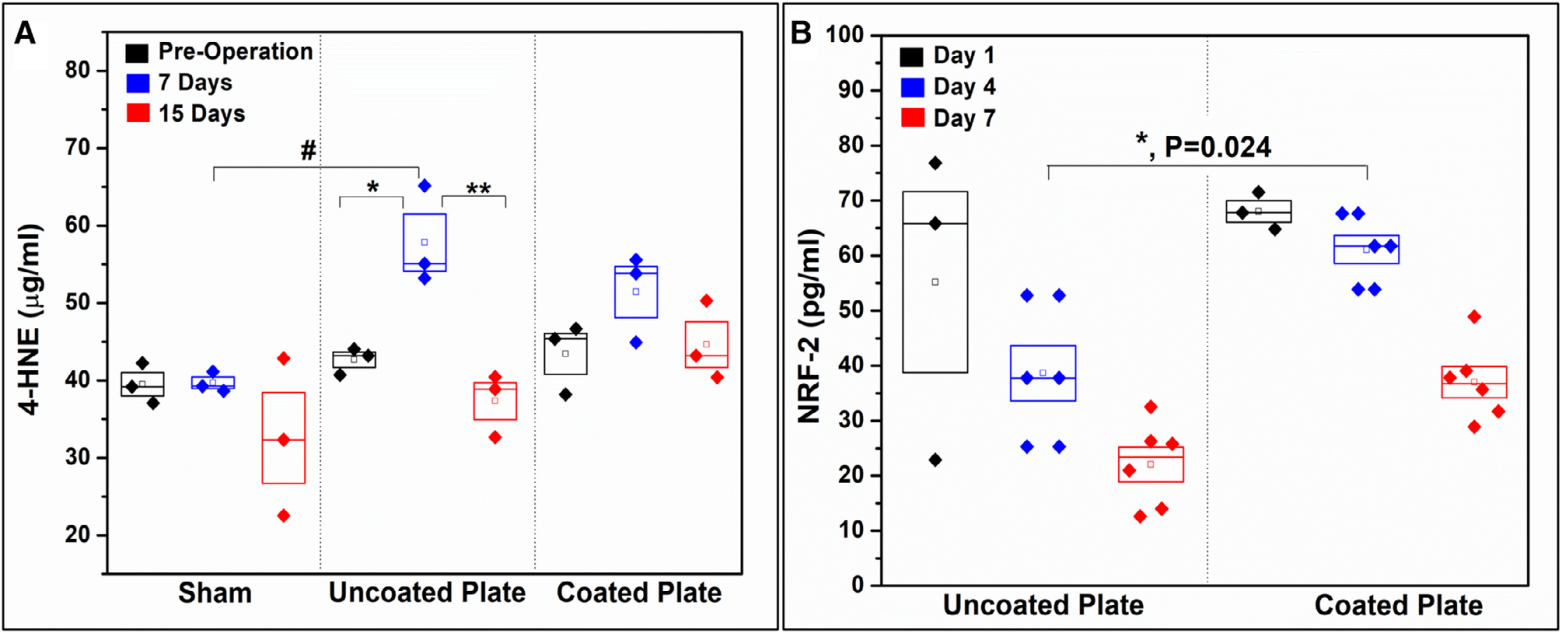
Bar graph shows 4‐HNE protein adduct concentration in the rat serum presurgery, 7 days postsurgery, and 15 days postsurgery (competitive ELISA). *p < 0.05 (one‐way ANOVA—Tukey's pairwise within the uncoated “bare” implant group) and #*p* < 0.05 (one‐way ANOVA—Tukey's pairwise, same time point different groups).

The bare silicon wafer control group showed a significant elevation in the 4‐HNE serum concentration at 7 days after surgery (57.81 ± 6.4 μg/mL) as compared with the preoperative levels (42.64 ± 1.72 μg/mL), with a reduction at 15 days after surgery (37.32 ± 4.11 μg/mL, *p* < 0.05). The 4‐HNE serum concentration for the uncoated “bare” implant group was significantly higher at 7 days as compared with the sham group (39.67 ± 1.28 μg/mL, *p* < 0.05). The sham and SiONPx‐coated plate groups did not show any significant differences among the three time points within the same group (*p* > 0.05; Fig. 7*A*). We also saw significant increased NRF2 activity in serum for the SiONPx‐coated group as compared with the bare implant group (Fig. 7*B*).

#### Histological analysis of harvested samples

##### Sanderson's staining

**Fig 8:**
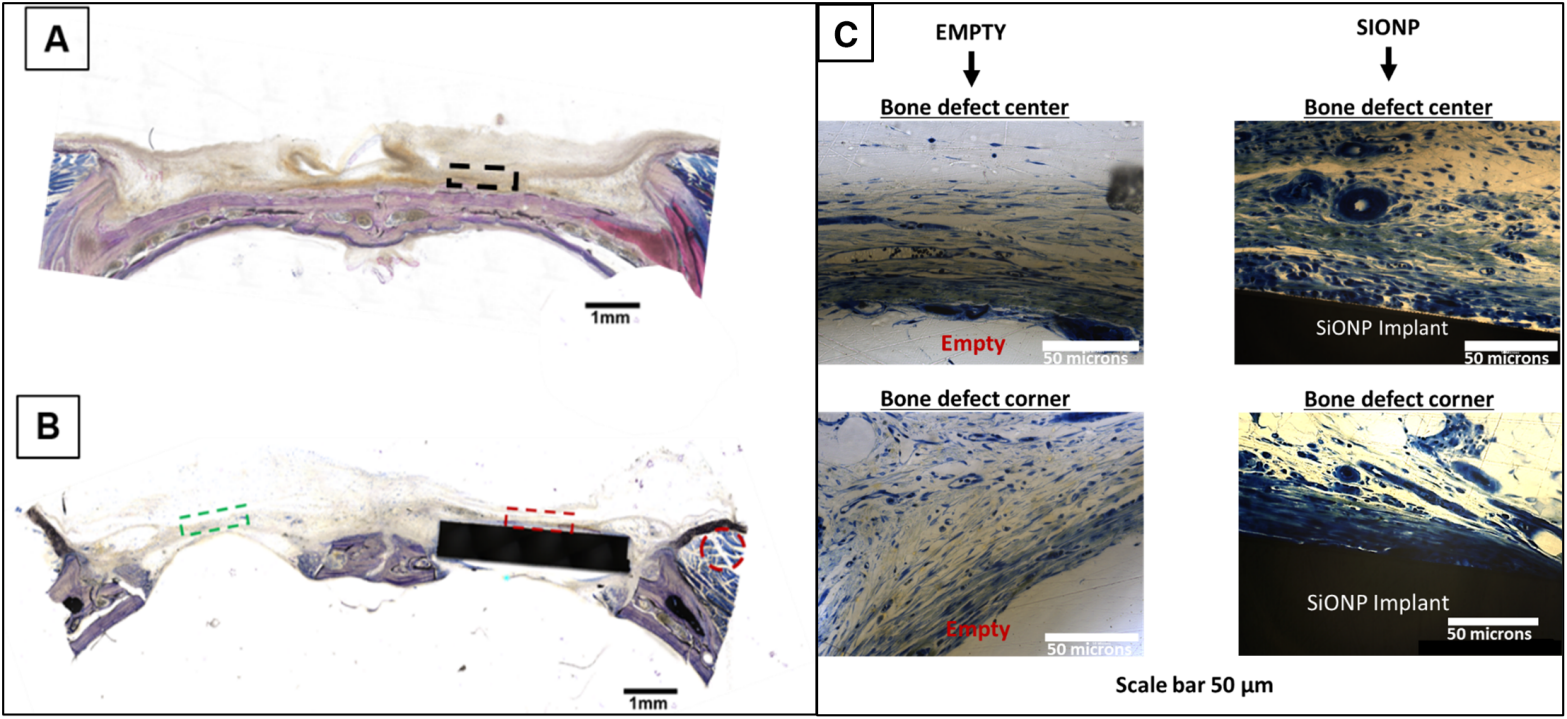
Bright field images acquired using the BIOQUANT Osteoimager showing coronal sections of rat calvaria after Sanderson's staining. Dotted rectangles represent the areas used for capturing immunofluorescent images. (*A*) The dotted black rectangular box is representative of a sham‐operated animal. (*B*) The dotted green rectangular box indicates the location assessed for the empty standard‐size calvarial defect, while the dotted red rectangular box indicates the location of the assessed implant‐filled standard‐size calvarial defect. The muscle is traced with a red circle. Scale bar = 1 mm. (*C*) Higher magnification images of empty defect (showing no vascular tissue formation) and coated implant surfaces (showing new vascular tissue formation). Uncoated surfaces did not exhibit any new vascular tissue formation, which matched that of empty defects. SiONP = silicon oxynitrophosphide.

Sanderson's staining of coronally sectioned calvaria is shown in Fig. 8 and shows areas used for capturing immunofluorescent images for CD31 and 4‐HNE. The coronal section of the sham‐operated group is seen in Fig. 8*A* where the dotted black rectangular box represents the analyzed area. The surgical bone defect sample is shown in Fig. 8*B*, where the dotted green rectangular box represents the empty defect, the dotted red rectangular box represents the new tissue formed on the plates, and the dotted red circle represents the rat calvarial muscle area analyzed. Fig. 8*C* shows high magnification images of the cross section, where there is more vascular as well as osteoid formation on the SiONPx surface as compared to the empty defect.

##### Immunostaining (CD31, ANG1, HIF‐1α, NRF2, and 4‐HNE)

**Fig 9:**
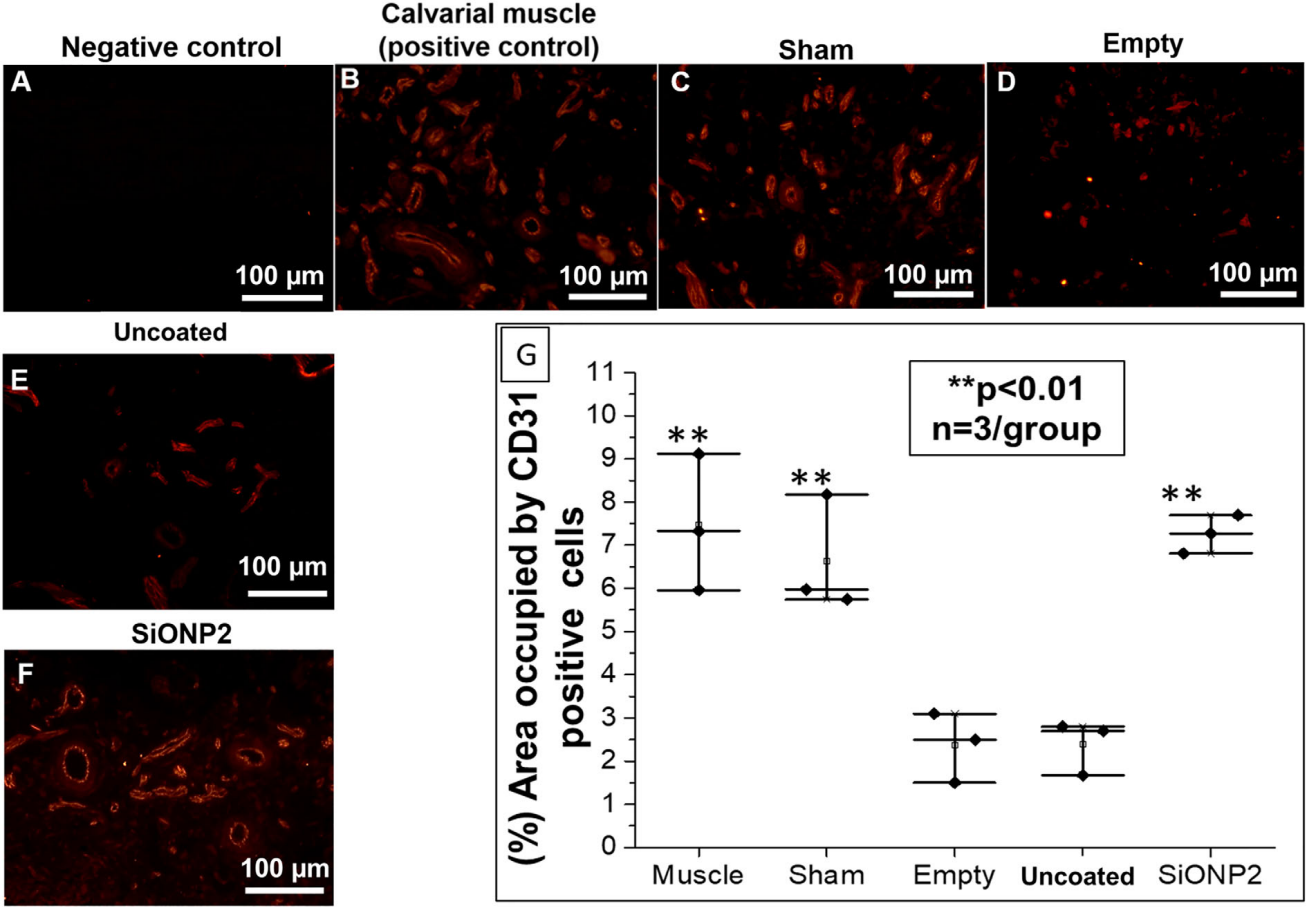
Immunofluorescence staining (Alexa Fluor 594) for CD31 15 days after implantation. Scale bar = 100 μm. (*A*) Negative staining control (no secondary antibody was used). (*B*) Calvarial muscle (positive control). (*C*) Sham (surgical procedure, no bone defects). (*D*) Empty defect. (*E*) Uncoated “bare” implant. (*F*) SiONP2. (*G*) Percentage area occupied by blood vessels and capillary network relative to sham. SiONP = silicon oxynitrophosphide. **p < 0.01, compared with empty and uncoated “bare” implant groups.

CD31 was used to compare samples collected from calvarial muscle, sham, empty, uncoated “bare” implant, and SiONPx2. And in the 4‐HNE analyses, samples from empty, sham, uncoated implant, and SiONPx2 were compared. Rats implanted with SiONPx showed significantly more CD31‐positive cells (twofold increase), than the empty and uncoated implant (*p* < 0.01) groups. There was no significant difference when quantified among SiONPx2, muscle (positive control), and sham groups (*p* > 0.05; Fig. 9).

**Fig 10:**
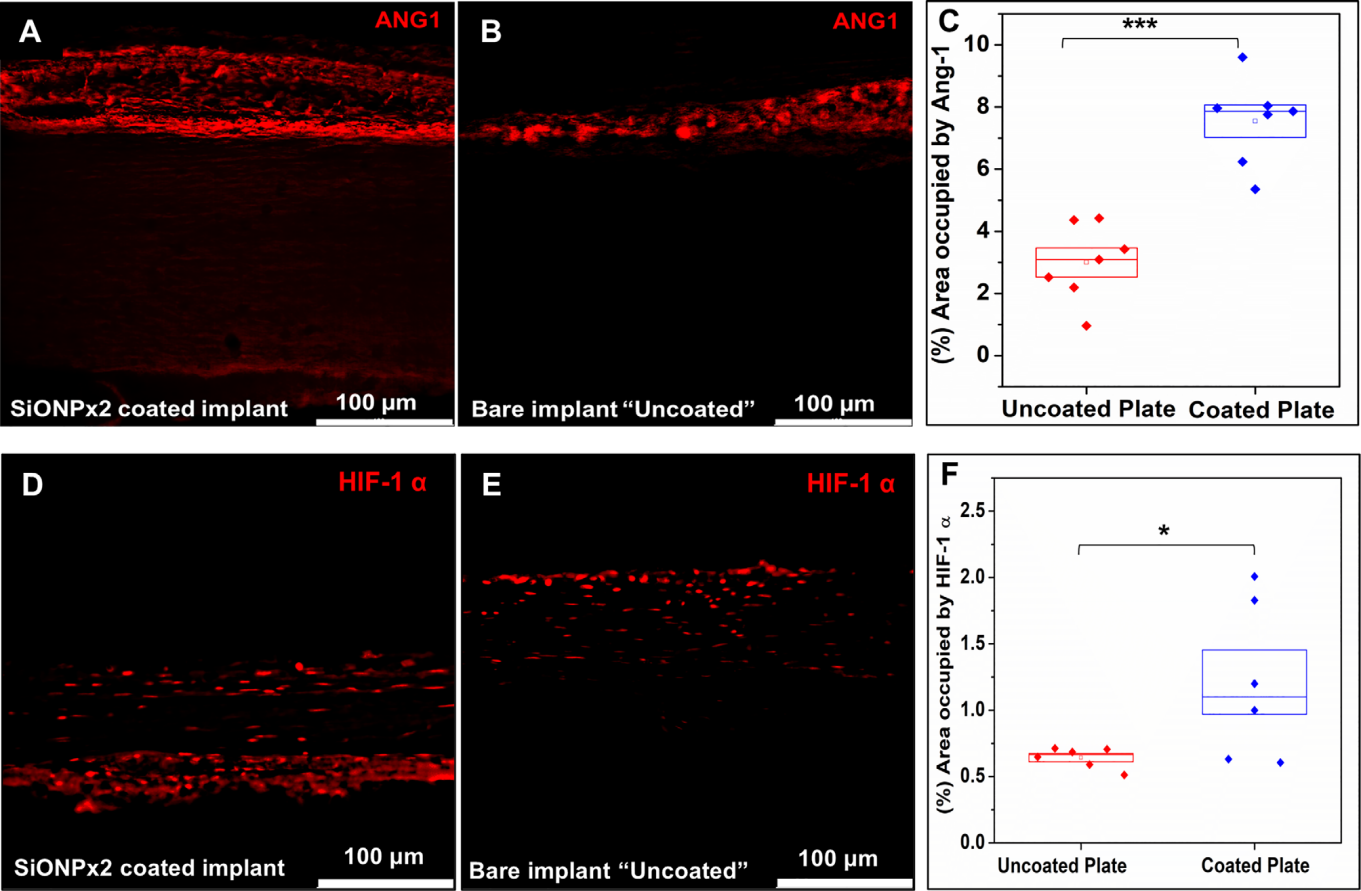
Immunofluorescence staining (Alexa Fluor 594) for Ang‐1 and HIF‐1α, 15 days after implantation. Scale bar 100 μm. (*A*) SiONPx2. (*B*) Uncoated “bare” implant. (*C*) Percentage of area occupied by Ang‐1. Statistical difference, ***p < 0.001. (*D*) SiONPx2. (*E*) Uncoated “bare” implant. (*F*) Percentage of area occupied by HIF‐1α. Statistical difference, *p < 0.05. Ang‐1 = Angiopoietin‐1; HIF‐1α = hypoxia inducible factor‐1 alpha; SiONP = silicon oxynitrophosphide.

ANG1 immunostaining (Fig. 10*A*–*C*) showed significantly higher expression of ANG1 on the SiONPx2‐coated surface as compared with the uncoated group. There was a significant difference of 2.5‐fold between the coated and uncoated group (*p* < 0.001). Fig. 10(*D*–*F*) shows the expression of HIF‐1α on the SiONPx‐coated and uncoated implant plates. Results show a significant difference (*p* < 0.05) between the two groups.

**Fig 11:**
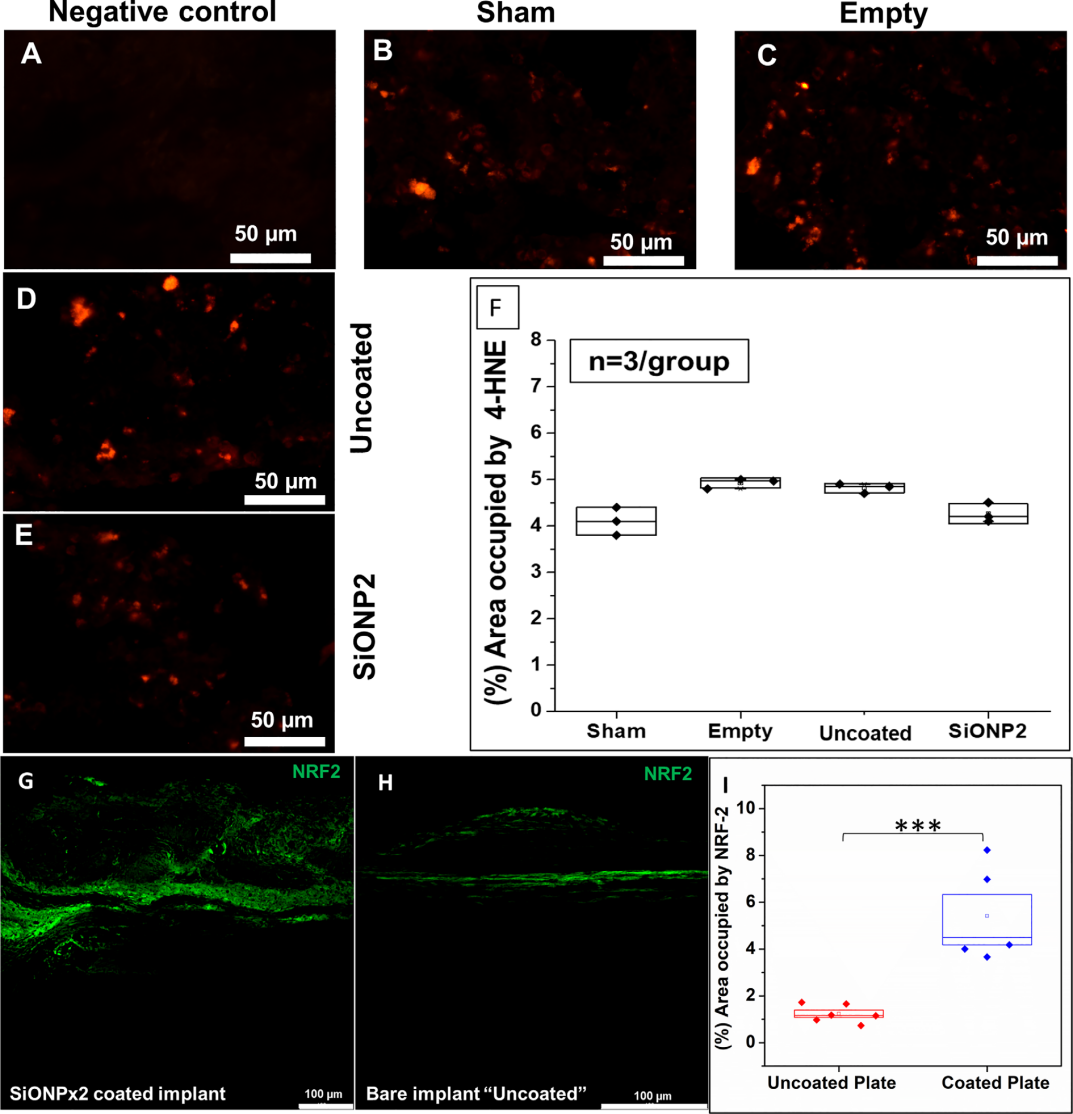
Immunofluorescence staining (Alexa Fluor 594) for 4‐HNE and (Alexa Fluor 488) for NRF‐2, 15 days after implantation. Scale bar = 50 μm. (*A*) Negative control. (*B*) Sham. (*C*) Empty defect. (*D*) Uncoated “bare” implant. (*E*) SiONP2. (*F*) Percentage of area occupied by 4‐HNE. After one‐way ANOVA—Tukey's pairwise, it was not detected difference among the studied groups (*p* > 0.05). (*G*) SiONPx2. (*H*) Bare implant. (*I*) Percentage of area occupied by NRF2. 4‐HNE = 4‐Hydroxynonenal; NRF2 = nuclear factor erythroid 2‐related factor 2; SiONP = silicon oxynitrophosphide. Statistical difference, ***p < 0.001.

The percentage of area occupied by 4‐HNE tissue fluorescence signal shows no statistical difference among the groups (*p* > 0.05; Fig. 11*A*–*F*). The tissues surrounding the SiONPx‐coated plate showed two times more NRF2 expression (Fig. 11*G*–*I*; significant difference) as compared with the uncoated plate group.

## Discussion

We found that SiONx and SiONPx groups could reduce cell death in HUVECs and improve proliferation (as seen in Fig. 1). The released ions and/or the surface energy seem to have a protective effect on HUVECs, even when exposed to 0.6mM H_2_O_2_. We could not find studies that correlate SiONx‐ or SiONPx‐based materials with a reduction of endothelial cell death, particularly under oxidative stress conditions. However, studies have reported that ionic silicon and silica‐based materials improve endothelial cells' proliferation by upregulating several angiogenic markers.^(^ ^11^, ^38^, ^49^, ^50^ ^)^ Our findings suggest that these biomaterials could accelerate tissue regeneration under unfavorable conditions, such as hypoxia and elevated ROS present in large and complex bone defects.

In the following in vitro experiments, we detected a significant improvement in the length of the capillary tubule network and the fibronectin deposition in all PECVD‐coated SiONx‐ and SiONPx‐implant plates, particularly with the SiONPx2 (O: 14.2 at %) group (Fig. 2*A*). Some previous studies have shown silica‐based materials can improve endothelial cells' capillary tubule formation.^(^ ^11^, ^38^ ^)^ However, none of these studies showed this effect under oxidative stress conditions, as observed in our experiments. Fibronectin is a glycoprotein that is produced by HUVECs and plays a major role in extracellular matrix formation during angiogenesis. This protein regulates vascular remodeling, endothelial cell migration, survival, and elongation.^(^ ^28^, ^51^, ^52^ ^)^ In Fig. 2*C*–*E*, we can see that SiONx, SiONPx1, and SiONPx2 groups show enhanced fibronectin deposition in a circular shape with significantly higher density in the SiONPx groups.

We found that ANG1 production was enhanced in HUVECs exposed to SiONx, SiONPx1, and SiONPx2 (Fig. 4*A*). In addition, all PECVD‐coated groups presented a significant reduction of 4‐HNE adduct (Fig. 4*B*). ANG1 has been observed in previous studies to protect endothelial cells against apoptosis when they are under unfavorable survival conditions.^(^ ^15^, ^17^ ^)^ Moreover, this protein is important for endothelial cell migration, proliferation, and differentiation.^(^ ^18^, ^53^ ^)^ Another pertinent aspect is that ANG1 can induce osteoblastic differentiation, bone matrix deposition, and enhance bone mineral density.^(^ ^54^, ^55^ ^)^ The 4‐HNE protein adduct is a product of lipid peroxidation and has been used to demonstrate oxidative stress levels in vivo and in vitro. Other molecules, such as malondialdehyde (MDA), have been used to measure oxidative stress levels; however, MDA is less stable with a shorter half‐life. Our results showed that the PECVD‐coated fixative implants reduced oxidative stress and enhanced ANG1 production, which could be preventing cell death as observed in our initial in vitro experiment (Fig. 1).

The evaluation of angiogenic gene expression markers showed that the mRNA levels of ANG1 were at least 2.5‐fold enhanced in the SiONPx groups, and that finding corroborates with the protein level in the conditioned medium. VEGFA and NES2 mRNA levels were also enhanced in all groups with SiONx and SiONPx. VEGFA is a well‐known major regulator of angiogenesis and can be stimulated by multiple factors.^(^ ^56^, ^57^ ^)^ Studies have shown that ionic silicon released from the mesoporous silica and bioactive glasses can enhance angiogenesis by upregulating VEGFA.^(^ ^11^, ^58^ ^)^ As mentioned above, ANG1 is relevant in angiogenesis and osteogenesis.^(^ ^15^, ^17^, ^18^, ^54^, ^55^ ^)^ NES2 is a large multidomain protein that plays a dominant role in regulating endothelial cell shape and migration. This protein connects the nuclei to the cytoskeleton and regulates the architecture of both structures, controlling the angiogenic loop formation during precapillary tubular network organization.^(^ ^59^ ^)^

Previously, a study reported that products of dissolution of calcium silicate can stimulate angiogenesis by inducing NOS upregulation.^(^ ^60^ ^)^ NO can have an ambiguous role in angiogenesis under oxidative stress conditions. On one hand, high levels of superoxide can neutralize the angiogenic effect of NO by forming peroxynitrite, which is a highly toxic molecule.^(^ ^61^ ^)^ On the other hand, authors have reported that NO suppresses angiostatin, which is an inhibitor of angiogenesis.^(^ ^62^ ^)^ However, NOS‐3 and NO are associated with fracture healing modulation and are necessary for adequate bone formation,^(^ ^63^, ^64^ ^)^ which supports the use of SiONx and SiONPx in bone defects. Although these in vitro studies give us great insight on the various factors that may be involved in enhanced angiogenesis for SiONx‐ and SiONPx‐coated plates, they have a limitation: they cannot replicate the complex in vivo environment.

In the in vivo experiments we measured angiogenesis by CD31 and ANG1. We also measured NRF2 activity and oxidative stress by 4‐HNE immunofluorescent staining. In addition, 4‐HNE was measured from blood samples collected before and after surgery. The SiONPx2‐coated plate was used for the in vivo study as it presented the most relevant outcome in the in vitro experiments. SiONPx2 plates enhanced ANG1 expression and increased blood vessel density regenerated on the coating surface were similar to those seen in the evaluation of blood vessel density in cortical bone. In 2006, Kingsmill and colleagues studied the cortical vascular canals in human cranium and mandible based on quantitative calculations from digital backscattered electron images.^(^ ^65^ ^)^ They reported that the mandible (labial aspect of the midline) has a high number of blood vessel canals (mean, 7.2/mm^2^) and a canal area of 7.5% compared with the parietal bone that has less blood vessel canals (mean, 5.9/mm^2^) and canal area of 5.5%.^(^ ^65^ ^)^ These results also support our in vitro observations and show that these coated plates can support and enhance angiogenesis in critical‐size bone defects. 4‐HNE immunostaining could not detect a difference between groups, likely because of lipid peroxidation product elimination or the adduction to other molecules by day 15. Studies have found that 4‐HNE is unstable and can be difficult to detect after 10 days from the initial oxidative stress event.^(^ ^66^, ^67^ ^)^ After 7 days, the serum levels of 4‐HNE adduct showed that animals implanted with SiONPx2 (O: 14.2 at %) in the left calvarial bone defect presented no difference compared with preoperative levels. This observation suggests that the surfaces coated with SiONx and SiONPx by PECVD can reduce ROS and possibly improve and accelerate osseointegration of the biomaterial.

In general, all elements used in our study for the PECVD‐surface coating have been reported to facilitate angiogenesis.^(^ ^14^, ^68^, ^69^ ^)^ Among Si, N, and P, Si is the most studied and supported in enhancing angiogenesis.^(^ ^11^, ^37^, ^39^, ^70^, ^71^ ^)^ As shown in Table 2, EDS analysis shows that the difference between SiONx, SiONPx1, and SiONPx2 (O: 14.2 at %) groups is mainly represented by Si, O, and N at %, as P represents <1 at % of surface composition. However, the addition of P and the different N_2_O flow rate between SiONPx1 and SiONPx2 (Table 1) played a role in the elemental surface composition of these two coatings. It seems that the elevated silicon at % can be responsible for the SiONPx1 and SiONPx2 outcome in enhancing angiogenesis and reducing the oxidative stress found in our in vitro studies. In terms of ionic surface composition, the different results observed in the SiONPx1 (O: 7.3 at %) and SiONPx2 (O: 14.2 at %) groups can be attributed to oxygen content. The SiONPx2 group presented twice more oxygen than the SiONPx1. Furthermore, despite not being part of this study, we have also shown in a concurrent study that SiONPx2 presented significantly reduced wettability compared with SiONPx1, which also can justify enhanced angiogenic effect of surfaces coated with SiONPx2,^(^ ^19^ ^)^ as more hydrophilic surfaces enhance angiogenesis by improving cell attachment and proliferation.^(^ ^72^, ^73^ ^)^

Antioxidant markers also play an important role in mitigating the effects of excessive ROS and oxidative stress. At the end of in vitro experiments, we measured the mRNA level of antioxidant enzymes and NOS. Our results showed that SOD‐1 and CAT‐1 were significantly upregulated by PECVD‐coated surfaces. Moreover, NOS‐3 levels were significantly elevated in SiONPx groups. SOD‐1 is an enzyme that catalyzes the conversion of superoxide, the main ROS produced during cell metabolism, to H_2_O_2_. CAT‐1 is a potent antioxidant protein that accelerates the conversion of H_2_O_2_ in H_2_O and O_2._ Thus, the enhancement of SOD‐1 and CAT‐1 activity reduces oxidative stress and accordingly improves biocompatibility ad osseointegration.^(^ ^74^ ^)^ NOS‐3 is an enzyme that participates in NO synthesis and is important for angiogenesis and tissue regeneration.^(^ ^75^ ^)^ Our group has reported the important role of ionic silicon in SOD‐1 regulation within osteoblasts differentiated from MC3T3 cells.^(^ ^9^ ^)^

NRF2 is a latent protein that is a major transcriptional activator of genes coding for enzymatic antioxidants, such as CAT and SOD, as well as a major anti‐inflammatory signaling molecule that can be induced to express upon traumatic injury.^(^ ^76^ ^)^ A study showed that a Si‐rich diet could reduce oxidative stress in the liver of aged rats by neutralizing peroxides generated from high cholesterol diets in a similar manner as hydroxytyrosol.^(^ ^77^ ^)^ This study showed that SOD‐1, CAT, and NRF2 were able to recover to the nominal levels as that of rats not given cholesterol and showed the ability of Si ions to neutralize ROS and rescue normal function.^(^ ^77^ ^)^ Our results showed for the first time that all plates coated with amorphous SiONx or SiONPx upregulated NRF2. Perhaps this major regulator is playing a role in the antioxidant effect of SiONx and SiONPx in HUVECs. The SOD enzyme has a cationic site formed by zinc (Zn) and/or copper (Cu) that by electrostatic interaction facilitates the bond between the enzyme and superoxide (O_2_^−^).^(^ ^78^, ^79^, ^80^ ^)^ Using the same rationale, the Si^4+^ released from the plate coated with SiONx and SiONPx^(^ ^9^ ^)^ can reduce oxidative stress by interacting with superoxide and preventing interaction with lipids, DNA, and proteins.

Finally, we speculate on the effect of these surfaces on antioxidant metalo‐enzymes such as SOD1, CAT, and NRF2. Antioxidant metalo‐enzymes are activated by transition metal cations such as Cu^2+^, Zn^2+^, and Mg^2+^ because of their ability to present electrons in a third orbital state for reaction with the enzymes.^(^ ^81^, ^82^ ^)^ This is required to overcome the spin orbital restriction associated with enzyme activity for this family of enzymes.^(^ ^83^ ^)^ We have shown in prior work that SiONx and SiONPx induce spin orbit splitting in which 2p Si electrons transition to third theoretical orbitals when N is added to their structures.^(^ ^84^, ^85^ ^)^ We suggest that the added electron availability not only overcomes this spin restriction, the presence of the higher valance state Si^4+^ increases charge transfer to antioxidants as follows:

(1) $$M^{\left(n+1\right)+}/\text{SOD1}+2n{O_{2}}^{-}+2nH^{+}=M^{\left(n+1\right)+}/\text{SOD1}+nH_{2}O_{2}+nO_{2}$$

In Equation (1), M represents transition metal cations such as Cu, Zn, and Mn and *n* = 1, 2, or 3. Equation (1) presents the overall reaction that has two steps. One step is the cation is reduced as it reduces superoxide. The other step involves the oxidation of the cation as superoxide is reduced.^(^ ^82^ ^)^ This means that the cation acts as a catalyst and as such conserves charge. For Si, the increased valence state of Si undergoes more moles of superoxide reduction. Antioxidants such as glutathione peroxidase further dissociate the peroxide molecule into hydroxyl groups, which are further reduced by CAT.^(^ ^86^ ^)^ For NRF2, which anchored in the cytoplasm through binding to Keap1, Si ions may dissociate Keap1 within the cytosol to release NRF2, which then translocate into the nucleus as seen in Fig. 11*G*–*I*. We will further investigate these reactions using metabolomics analysis to better understand the reaction efficiency of such reactions in future work to further elucidate this reaction mechanism.

## Conclusion

In conclusion, our study found that fixative plates coated with SiONx and SiONPx sustained cellular viability, enhanced matrix deposition, and capillary tube formation in HUVECs under toxic oxidative stress. In addition, we found under the same conditions that these coatings upregulated ANG1, VEGFA, and antioxidant enzymes NRF2, SOD1, and CAT. An in vivo experiment showed that the plates coated with SiONx and SiONPx, mainly SiONPx2, enhanced angiogenesis and reduced oxidative stress. These findings support the use of fixative implants coated with SiONx and SiONPx formed by Si (58.7 at %), O (14.2 at %), N (26.8 at %), and P (0.27 at %) in bone defect by creating a favorable environment for faster healing rates, and accordingly mitigation of implant loosening and failure. The use of a large skeletally mature animal like pigs, dogs, goats or sheep will provide future clinical relevance and efficacy of these fixative implant coatings for further translational use. Finally, the PECVD manufacturing cost, automation during fabrication, ability to coat all 3D and porous geometries, and relatively rapid manufacturing time make these nanoscale coatings highly viable for potential future clinical use.

## Disclosures

The authors declare no conflict of interest.

## Author Contributions

**Felipe Monte:** Data curation; formal analysis; methodology; software; validation; visualization; writing‐original draft. **Neelam Ahuja:** Formal analysis; investigation; methodology; validation; writing‐original draft. **Kamal Awad:** Formal analysis; investigation; software; writing‐original draft; writing‐review and editing. **Zui Pan:** Resources. **Simon Young:** Conceptualization; writing‐review and editing. **Harry Kim:** Conceptualization; resources; supervision; writing‐review and editing. **Pranesh Aswath:** Resources; supervision; writing‐review and editing. **Marco Brotto:** Conceptualization; resources; supervision; writing‐review and editing. **Venu G Varanasi:** Funding acquisition; project administration; resources; supervision; writing‐review and editing.

### Peer Review

The peer review history for this article is available at https://publons.com/publon/10.1002/jbm4.10425.

## References

[1] Vanhegan IS , Malik AK , Jayakumar P , Ul Islam S , Haddad FS . A financial analysis of revision hip arthroplasty. J Bone Joint Surg Br. 2012;94(5):619–23. 22529080 10.1302/0301-620X.94B5.27073

[2] Pietropaoli D , Ortu E , Severino M , Ciarrocchi I , Gatto R , Monaco A . Glycation and oxidative stress in the failure of dental implants: a case series. BMC Res Notes. 2013;6:296. 23890159 10.1186/1756-0500-6-296 PMC3733866

[3] Lindhe J , Meyle J , Group D of European Workshop on Periodontology . Peri‐Implant Diseases: consensus report of the Sixth European Workshop on Periodontology. J Clin Periodontol. 2008;35(8 suppl):282–5. 18724855 10.1111/j.1600-051X.2008.01283.x

[4] Szpalski C , Barr J , Wetterau M , Saadeh PB , Warren SM . Cranial bone defects: current and future strategies. Neurosurg Focus. 2010;29(6):E8. 10.3171/2010.9.FOCUS10201 21121722

[5] Liu Z , Liu Y , Xu Q , et al. Critical role of vascular peroxidase 1 in regulating endothelial nitric oxide synthase endothelial nitric oxide synthase nitric oxide asymmetricdimethylarginine angiotensin II oxidative stress. Redox Biol. 2017;12:226–32. 28264790 10.1016/j.redox.2017.02.022 PMC5338721

[6] Saran U , Piperni SG , Chatterjee S . Role of angiogenesis in bone repair. Arch Biochem Biophys. 2014;561:109–17. 25034215 10.1016/j.abb.2014.07.006

[7] Bock RM , McEntire BJ , Bal BS , Rahaman MN , Boffelli M , Pezzotti G . Surface modulation of silicon nitride ceramics for orthopaedic applications. Acta Biomater. 2015;26:318–30. 26302831 10.1016/j.actbio.2015.08.014

[8] Ilyas A , Lavrik NV , Kim HKW , Aswath PB , Varanasi VG . Enhanced interfacial adhesion and osteogenesis for rapid “bone‐like” biomineralization by PECVD‐based silicon oxynitride overlays. ACS Appl Mater Interfaces. 2015;7(28):15368–79. 26095187 10.1021/acsami.5b03319 PMC6508966

[9] Ilyas A , Odatsu T , Shah A , et al. Amorphous silica: a new antioxidant role for rapid critical‐sized bone defect healing. Adv Healthc Mater. 2016;5(17):2199–213. 27385056 10.1002/adhm.201600203 PMC6635139

[10] Awad KR , Ahuja N , Shah A , et al. Silicon nitride enhances osteoprogenitor cell growth and differentiation via increased surface energy and formation of amide and nanocrystalline HA for craniofacial reconstruction. Med Devices Sens. 2019;2(2):e10032. 10.1002/mds3.10032 PMC9248716 35781939

[11] Dashnyam K , Jin G‐Z , Kim J‐H , Perez R , Jang J‐H , Kim H‐W . Promoting angiogenesis with mesoporous microcarriers through a synergistic action of delivered silicon ion and VEGF. Biomaterials. 2017;116:145–57. 27918936 10.1016/j.biomaterials.2016.11.053

[12] Camalier CE , Yi M , Yu L‐RR , et al. An integrated understanding of the physiological response to elevated extracellular phosphate. J Cell Physiol. 2013;228(7):1536–50. 23280476 10.1002/jcp.24312 PMC3702686

[13] Lin Y , Mckinnon KE , Ha SW , Beck GR . Inorganic phosphate induces cancer cell mediated angiogenesis dependent on forkhead box protein C2 (FOXC2) regulated osteopontin expression. Mol Carcinog. 2015;54(9):926–34. 24700685 10.1002/mc.22153 PMC4183733

[14] Saghiri MA , Asatourian A , Orangi J , Sorenson CM , Sheibani N . Functional role of inorganic trace elements in angiogenesis—Part I: N, Fe, Se, P, Au, and Ca. Crit Rev Oncol Hematol. 2015;96:129–42. 26088454 10.1016/j.critrevonc.2015.05.010

[15] Harfouche R , Hasséssian HM , Guo Y , et al. Mechanisms which mediate the antiapoptotic effects of angiopoietin‐1 on endothelial cells. Microvasc Res. 2002;64(1):135–47. 12074640 10.1006/mvre.2002.2421

[16] Abdel‐Malak NA , Mofarrahi M , Mayaki D , Khachigian LM , Hussain SNA . Early growth response‐1 regulates angiopoietin‐1‐induced endothelial cell proliferation, migration, and differentiation. Arterioscler Thromb Vasc Biol. 2009;29(2):209–16. 19112164 10.1161/ATVBAHA.108.181073

[17] Jin Kwak H , So J‐N , Jae Lee S , Kim I , Koh GY . Angiopoietin‐1 is an apoptosis survival factor for endothelial cells. FEBS Lett. 1999;448(2–3):249–53. 10218485 10.1016/s0014-5793(99)00378-6

[18] Harel S , Mayaki D , Sanchez V , Hussain SNAA . NOX2, NOX4, and mitochondrial‐derived reactive oxygen species contribute to angiopoietin‐1 signaling and angiogenic responses in endothelial cells. Vascul Pharmacol. 2017;92:22–32. 28351775 10.1016/j.vph.2017.03.002

[19] Monte F , Awad KR , Ahuja N , et al. Amorphous silicon oxynitrophosphide coated implants boost angiogenic activity of endothelial cells. Tissue Eng Part A. 2020;26(1–2):15–27. 31044666 10.1089/ten.tea.2019.0051 PMC6983748

[20] Monte F , Cebe T , Ripperger D , et al. Ionic silicon improves endothelial cells' survival under toxic oxidative stress by overexpressing angiogenic markers and antioxidant enzymes. J Tissue Eng Regen Med. 2018;12(11):2203–20. 30062712 10.1002/term.2744 PMC6508967

[21] Sui X , Xu Z , Xie M , Pei D . Resveratrol inhibits hydrogen peroxide‐induced apoptosis in endothelial cells via the activation of PI3K/Akt by miR‐126. J Atheroscler Thromb. 2014;21(2):108–18. 24107596 10.5551/jat.19257

[22] He B , Fu G , Du X , Chu H . Halofuginone protects HUVECs from H_2_O_2_ ‐induced injury by modulating VEGF/JNK signaling pathway. J Chin Med Assoc. 2019;82(2):92–8. 30839497 10.1097/JCMA.0000000000000008

[23] Wu J , Lei Z , Yu J . Hypoxia induces autophagy in human vascular endothelial cells in a hypoxia‐inducible factor 1‐dependent manner. Mol Med Rep. 2015;11(4):2677–82. 25514934 10.3892/mmr.2014.3093

[24] Lim YC , McGlashan SR , Cooling MT , Long DS . Culture and detection of primary cilia in endothelial cell models. Cilia. 2015;4:11. 26430510 10.1186/s13630-015-0020-2 PMC4590708

[25] Kocherova I , Bryja A , Mozdziak P , et al. Human umbilical vein endothelial cells (HUVECs) co‐culture with osteogenic cells: from molecular communication to engineering prevascularised bone grafts. J Clin Med. 2019;8(10):1602. 10.3390/jcm8101602 PMC6832897 31623330

[26] Ferreira T , Rasband W . ImageJ user guide. Version 1.46r. Bethesda, MD: National Institutes of Health;. 2012.

[27] Chen Y , Yu Q , Xu CB . A convenient method for quantifying collagen fibers in atherosclerotic lesions by imagej software. Int J Clin Exp Med. 2017;10(10):14904–10.

[28] Hielscher A , Ellis K , Qiu C , Porterfield J , Gerecht S . Fibronectin deposition participates in extracellular matrix assembly and vascular morphogenesis. PLoS One. 2016;11(1):1–27. 10.1371/journal.pone.0147600 PMC4728102 26811931

[29] Zhu Y , Zhang Y‐J , Liu W‐W , Shi A‐W , Gu N . Salidroside suppresses HUVECs cell injury induced by oxidative stress through activating the Nrf2 signaling pathway. Molecules. 2016;21(8):1033. 10.3390/molecules21081033 PMC6273208 27517893

[30] Wei D‐H , Deng J‐L , Shi R‐Z , et al. Epimedin C protects H_2_O_2_‐induced peroxidation injury by enhancing the function of endothelial progenitor HUVEC populations. Biol Pharm Bull. 2019;42(9):1491–9. 31204351 10.1248/bpb.b19-00159

[31] Gao S , Li S , Li Q , et al. Protective effects of salvianolic acid B against hydrogen peroxide‐induced apoptosis of human umbilical vein endothelial cells and underlying mechanisms. Int J Mol Med. 2019;44(2):457–68. 31173197 10.3892/ijmm.2019.4227 PMC6605496

[32] Reuter S , Gupta SC , Chaturvedi MM , Aggarwal BB . Oxidative stress, inflammation, and cancer: how are they linked? Free Radic Biol Med. 2010;49(11):1603–16. 20840865 10.1016/j.freeradbiomed.2010.09.006 PMC2990475

[33] Jia C‐H , Li M , Liu J , et al. IKK‐β mediates hydrogen peroxide induced cell death through p85 S6K1. Cell Death Differ. 2013;20(2):248–58. 22955948 10.1038/cdd.2012.115 PMC3554328

[34] Cui W , Leng B , Liu W , Wang G . Suppression of apoptosis in human umbilical vein endothelial cells (HUVECs) by klotho protein is associated with reduced endoplasmic reticulum oxidative stress and activation of the PI3K/AKT pathway. Med Sci Monit. 2018;24:8489–99. 30471224 10.12659/MSM.911202 PMC6270887

[35] Clonetics Endothelial Cell System [Internet]. 2017. p. 1–15. Available from: https://www.lonza.com.

[36] Arnaoutova I , Kleinman HK . In vitro angiogenesis: endothelial cell tube formation on gelled basement membrane extract. Nat Protoc. 2010;5(4):628–35. 20224563 10.1038/nprot.2010.6

[37] Keshaw H , Forbes A , Day RM . Release of angiogenic growth factors from cells encapsulated in alginate beads with bioactive glass. Biomaterials. 2005;26(19):4171–9. 15664644 10.1016/j.biomaterials.2004.10.021

[38] Zhai W , Lu H , Chen L , et al. Silicate bioceramics induce angiogenesis during bone regeneration. Acta Biomater. 2012;8(1):341–9. 21964215 10.1016/j.actbio.2011.09.008

[39] Schmitz JP , Hollinger JO . The critical size defect as an experimental model for craniomandibulofacial nonunions. Clin. Orthop. Relat. Res. 1986;205:299–308. 3084153

[40] Robinson NB , Krieger K , Khan FM , et al. The current state of animal models in research: a review. Int J Surg. 2019;72:9–13. 31627013 10.1016/j.ijsu.2019.10.015

[41] Gomes PS , Fernandes MH . Rodent models in bone‐related research: the relevance of calvarial defects in the assessment of bone regeneration strategies. Lab Anim. 2011;45(1):14–24. 21156759 10.1258/la.2010.010085

[42] McGovern JA , Griffin M , Hutmacher DW . Animal models for bone tissue engineering and modelling disease. Dis Model Mech. 2018;11(4):1–14. 10.1242/dmm.033084 PMC5963860 29685995

[43] Garcia P , Histing T , Holstein J , et al. Rodent animal models of delayed bone healing and non‐union formation: a comprehensive review. Eur Cells Mater. 2013;26:1–14. 10.22203/ecm.v026a01 23857280

[44] Cooper GM , Mooney MP , Gosain AK , Campbell PG , Losee JE , Huard J . Testing the critical size in calvarial bone defects: revisiting the concept of a critical‐size defect. Plast Reconstr Surg. 2010;125(6):1685–92. 20517092 10.1097/PRS.0b013e3181cb63a3 PMC2946111

[45] Porto GG , Porto GG , Vasconcelos BC , et al. Is a 5 mm rat calvarium defect really critical? Acta Cir Bras. 2012;27(11):757–60. 23117606 10.1590/s0102-86502012001100003

[46] Lee JY , Musgrave D , Pelinkovic D , et al. Effect of bone morphogenetic protein‐2‐expressing muscle‐derived cells on healing of critical‐sized bone defects in mice. J Bone Joint Surg Am. 2001;83(7):1032–9. 11451972 10.2106/00004623-200107000-00008

[47] Chatzipetros E , Christopoulos P , Donta C , et al. Application of nano‐hydroxyapatite/chitosan scaffolds on rat calvarial critical‐sized defects: a pilot study. Med Oral Patol Oral Cir Bucal. 2018;23(5):e625. 30148464 10.4317/medoral.22455 PMC6167094

[48] Akkiraju H , Bonor J , Nohe A . An improved Immunostaining and imaging methodology to determine cell and protein distributions within the bone environment. J Histochem Cytochem. 2016;64(3):168–78. 26718242 10.1369/0022155415626765 PMC4810797

[49] Li H , Chang J . Bioactive silicate materials stimulate angiogenesis in fibroblast and endothelial cell co‐culture system through paracrine effect. Acta Biomater. 2013;9(6):6981–91. 23416471 10.1016/j.actbio.2013.02.014

[50] Dashnyam K , El‐Fiqi A , Buitrago JO , Perez RA , Knowles JC , Kim H‐W . A mini review focused on the proangiogenic role of silicate ions released from silicon‐containing biomaterials. J Tissue Eng. 2017;8:204173141770733. 10.1177/2041731417707339 PMC5435366 28560015

[51] Chiang HY , Korshunov VA , Serour A , Shi F , Sottile J . Fibronectin is an important regulator of flow‐induced vascular remodeling. Arterioscler Thromb Vasc Biol. 2009;29(7):1074–9. 19407246 10.1161/ATVBAHA.108.181081 PMC3091823

[52] Kiyonaga H , Doi Y , Karasaki Y , Arashidani K , Itoh H , Fujimoto S . Expressions of endothelin‐1, fibronectin, and interleukin‐1alpha of human umbilical vein endothelial cells under prolonged culture. Med Electron Microsc. 2001;34(1):41–53. https://orcid.org/10.1007/s007950100003. PMID: 11479772. 11479772 10.1007/s007950100003

[53] Abdel‐Malak NA , Srikant CB , Kristof AS , Magder SA , Di Battista JA , Hussain SNA . Angiopoietin‐1 promotes endothelial cell proliferation and migration through AP‐1‐dependent autocrine production of interleukin‐8. Blood. 2008;111(8):4145–54. 18252863 10.1182/blood-2007-08-110338

[54] Suzuki T , Miyamoto T , Fujita N , et al. Osteoblast‐specific Angiopoietin 1 overexpression increases bone mass. Biochem Biophys Res Commun. 2007;362(4):1019–25. 17825261 10.1016/j.bbrc.2007.08.099

[55] Park S‐H , Lee J , Kang M‐A , et al. Potential of l‐thyroxine to differentiate osteoblast‐like cells via Angiopoietin1. Biochem Biophys Res Commun. 2016;478(3):1409–15. 27569283 10.1016/j.bbrc.2016.08.137

[56] Ferrara N , Gerber H‐P , LeCouter J . The biology of VEGF and its receptors. Nat Med. 2003;9(6):669–76. 12778165 10.1038/nm0603-669

[57] Shibuya M . Vascular endothelial growth factor and its receptor system: physiological functions in angiogenesis and pathological roles in various diseases. J Biochem. 2013;153(1):13–9. 23172303 10.1093/jb/mvs136 PMC3528006

[58] Zhao S , Li L , Wang H , et al. Wound dressings composed of copper‐doped borate bioactive glass microfibers stimulate angiogenesis and heal full‐thickness skin defects in a rodent model. Biomaterials. 2015;53:379–91. 25890736 10.1016/j.biomaterials.2015.02.112

[59] King SJ , Nowak K , Suryavanshi N , Holt I , Shanahan CM , Ridley AJ . Nesprin‐1 and nesprin‐2 regulate endothelial cell shape and migration. Cytoskeleton. 2014;71(7):423–34. 24931616 10.1002/cm.21182

[60] Chou MY , Kao CT , Hung CJ , et al. Role of the P38 pathway in calcium silicate cement‐induced cell viability and angiogenesis‐related proteins of human dental pulp cell in vitro. J Endod. 2014;40(6):818–24. 24862709 10.1016/j.joen.2013.09.041

[61] Förstermann U , Sessa WC . Nitric oxide synthases: regulation and function. Eur. Heart J. 2012;33(7):829–37. 21890489 10.1093/eurheartj/ehr304 PMC3345541

[62] Matsunaga T , Weihrauch DW , Moniz MC , Tessmer J , Warltier DC , Chilian WM . Angiostatin inhibits coronary angiogenesis during impaired production of nitric oxide. Circulation. 2002;105(18):2185–91. 11994253 10.1161/01.cir.0000015856.84385.e9

[63] Diwan AD , Wang MINX , Jang D , Zhu WEI , Murrell GAC . Nitric oxide modulates fracture healing. J Bone Miner Res. 2000;15(2):342–51. 10703937 10.1359/jbmr.2000.15.2.342

[64] Zhu W , Diwan AD , Lin JH , Murrell GA . Nitric oxide synthase isoforms during fracture healing. J Bone Miner Res. 2001;16(3):535–40. 11277271 10.1359/jbmr.2001.16.3.535

[65] Kingsmill VJ , Gray CM , Moles DR , Boyde A . Cortical vascular canals in human mandible and other bones. J Dent Res. 2007 Apr;86(4):368–72. 17384034 10.1177/154405910708600413

[66] Kim DH , Kwack SJ , Yoon KS , Choi JS , Lee BM . 4‐Hydroxynonenal: a superior oxidative biomarker compared to malondialdehyde and carbonyl content induced by carbon tetrachloride in rats. J Toxicol Environ Health A. 2015;78(16):1051–62. 26252470 10.1080/15287394.2015.1067505

[67] Spickett CM . The lipid peroxidation product 4‐hydroxy‐2‐nonenal: advances in chemistry and analysis. Redox Biol. 2013;1(1):145–52. 24024147 10.1016/j.redox.2013.01.007 PMC3757682

[68] Saghiri MA , Asatourian A , Orangi J , Sorenson CM , Sheibani N . Functional role of inorganic trace elements in angiogenesis‐part II: Cr, Si, Zn, Cu, and S. Crit Rev Oncol Hematol. 2015;96(1):143–55. 26088455 10.1016/j.critrevonc.2015.05.011

[69] Saghiri MA , Orangi J , Asatourian A , Sorenson CM , Sheibani N , Saghiri MA . Functional role of inorganic trace elements in in angiogenesis part III: (Ti, Li, Ce, As, Hg, Va, Nb and Pb) HHS public access. Crit Rev Oncol Hematol. 2016;98:290–301. 26638864 10.1016/j.critrevonc.2015.10.004 PMC5223240

[70] Haro Durand LA , Vargas GE , Vera‐Mesones R , et al. In vitro human umbilical vein endothelial cells response to ionic dissolution products from lithium‐containing 45S5 bioactive glass. Materials (Basel). 2017;10(7):740. 10.3390/ma10070740 PMC5551783 28773103

[71] Day RM . Bioactive glass stimulates the secretion of angiogenic growth factors and angiogenesis in vitro. Tissue Eng. 2005;11(5–6):768–77. 15998217 10.1089/ten.2005.11.768

[72] Sarapirom S , Lee JS , Jin SB , et al. Wettability effect of PECVD‐SiOx films on poly(lactic acid) induced by oxygen plasma on protein adsorption and cell attachment. J Phys Conf Ser. 2013;423(1):12042.

[73] Arima Y , Iwata H . Effect of wettability and surface functional groups on protein adsorption and cell adhesion using well‐defined mixed self‐assembled monolayers. Biomaterials. 2007;28(20):3074–82. 17428532 10.1016/j.biomaterials.2007.03.013

[74] Mouthuy PA , Snelling SJB , Dakin SG , et al. Biocompatibility of implantable materials: an oxidative stress viewpoint. Biomaterials. 2016;109:55–68. 27669498 10.1016/j.biomaterials.2016.09.010

[75] Aicher A , Heeschen C , Mildner‐Rihm C , et al. Essential role of endothelial nitric oxide synthase for mobilization of stem and progenitor cells. Nat Med. 2003;9(11):1370–6. 14556003 10.1038/nm948

[76] Gallorini M , Petzel C , Bolay C , et al. Activation of the Nrf2‐regulated antioxidant cell response inhibits HEMA‐induced oxidative stress and supports cell viability. Biomaterials. 2015;56:114–28. 25934285 10.1016/j.biomaterials.2015.03.047

[77] Santos‐López JA , Garcimartín A , Merino P , et al. Effects of silicon vs. Hydroxytyrosol‐enriched restructured pork on liver oxidation status of aged rats fed high‐saturated/high‐cholesterol diets. PLoS One. 2016;11(1):e0147469. 26807847 10.1371/journal.pone.0147469 PMC4726576

[78] Desideri A , Polticelli F , Falconi M , et al. Electrostatic recognition in redox copper proteins: a 1H NMR study of the protonation behavior of His 19 in oxidized and reduced Cu,Zn superoxide dismutase. Arch Biochem Biophys. 1993;301(2):244–50. 8384828 10.1006/abbi.1993.1140

[79] Fisher CL , Hallewell RA , Roberts VA , Tainer JA , Getzoff ED . Probing the structural basis for enzyme‐substrate recognition in Cu,Zn superoxide dismutase. Free Radic Res Commun. 1991;12–13(Pt 1):287–96. 10.3109/10715769109145797 1649096

[80] Shi Y , Mowery RA , Shaw BF . Effect of metal loading and subcellular pH on net charge of superoxide dismutase‐1. J Mol Biol. 2013;425(22):4388–404. 23871896 10.1016/j.jmb.2013.07.018

[81] Hippeli S , Elstner EF . Transition metal ion‐catalyzed oxygen activation during pathogenic processes. FEBS Lett. 1999;443(1):1–7. 9928941 10.1016/s0014-5793(98)01665-2

[82] Brazier MW , Wedd AG , Collins SJ . Antioxidant and metal chelation‐based therapies in the treatment of prion disease. Antioxidants. 2014;3(2):288–308. 26784872 10.3390/antiox3020288 PMC4665489

[83] Riley PA . Free radicals in biology: oxidative stress and the effects of ionizing radiation. Int J Radiat Biol. 1994;65(1):27–33. 7905906 10.1080/09553009414550041

[84] Ilyas MA , Velton E , Shah A , et al. Rapid regeneration of vascularized bone by nanofabricated amorphous silicon. J Biomed Nanotechnol. 2019;15:1–15. 31072432 10.1166/jbn.2019.2779 PMC9841885

[85] Varanasi VG , Ilyas A , Velten MF , Shah A , Lanford WA , Aswath PB . Role of hydrogen and nitrogen on the surface chemical structure of bioactive amorphous silicon oxynitride films. J Phys Chem B. 2017;121(38):8991–9005. 28825836 10.1021/acs.jpcb.7b05885 PMC6542473

[86] Kurutas EB . The importance of antioxidants which play the role in cellular response against oxidative/nitrosative stress: current state. Nutr J. 2016;15(1):71. 27456681 10.1186/s12937-016-0186-5 PMC4960740

